# Regulatory sites for splicing in human basal ganglia are enriched for disease-relevant information

**DOI:** 10.1038/s41467-020-14483-x

**Published:** 2020-02-25

**Authors:** Sebastian Guelfi, Karishma D’Sa, Juan A. Botía, Jana Vandrovcova, Regina H. Reynolds, David Zhang, Daniah Trabzuni, Leonardo Collado-Torres, Andrew Thomason, Pedro Quijada Leyton, Sarah A. Gagliano Taliun, Mike A. Nalls, International Parkinson’s Disease Genomics Consortium (IPDGC), Alastair J. Noyce, Aude Nicolas, Mark R. Cookson, Sara Bandres-Ciga, J. Raphael Gibbs, Dena G. Hernandez, Andrew B. Singleton, Xylena Reed, Hampton Leonard, Cornelis Blauwendraat, Faraz Faghri, Jose Bras, Rita Guerreiro, Arianna Tucci, Demis A. Kia, Henry Houlden, Helene Plun-Favreau, Kin Y Mok, Nicholas W. Wood, Ruth Lovering, Lea R’Bibo, Mie Rizig, Viorica Chelban, Manuela Tan, Huw R. Morris, Ben Middlehurst, John Quinn, Kimberley Billingsley, Peter Holmans, Kerri J. Kinghorn, Patrick Lewis, Valentina Escott-Price, Nigel Williams, Thomas Foltynie, Alexis Brice, Fabrice Danjou, Suzanne Lesage, Jean-Christophe Corvol, Maria Martinez, Anamika Giri, Claudia Schulte, Kathrin Brockmann, Javier Simón-Sánchez, Peter Heutink, Thomas Gasser, Patrizia Rizzu, Manu Sharma, Joshua M. Shulman, Laurie Robak, Steven Lubbe, Niccolo E. Mencacci, Steven Finkbeiner, Codrin Lungu, Sonja W. Scholz, Ziv Gan-Or, Guy A. Rouleau, Lynne Krohan, Jacobus J. van Hilten, Johan Marinus, Astrid D. Adarmes-Gómez, Inmaculada Bernal-Bernal, Marta Bonilla-Toribio, Dolores Buiza-Rueda, Fátima Carrillo, Mario Carrión-Claro, Pablo Mir, Pilar Gómez-Garre, Silvia Jesús, Miguel A. Labrador-Espinosa, Daniel Macias, Laura Vargas-González, Carlota Méndez-del-Barrio, Teresa Periñán-Tocino, Cristina Tejera-Parrado, Monica Diez-Fairen, Miquel Aguilar, Ignacio Alvarez, María Teresa Boungiorno, Maria Carcel, Pau Pastor, Juan Pablo Tartari, Victoria Alvarez, Manuel Menéndez González, Marta Blazquez, Ciara Garcia, Esther Suarez-Sanmartin, Francisco Javier Barrero, Elisabet Mondragon Rezola, Jesús Alberto Bergareche Yarza, Ana Gorostidi Pagola, Adolfo López de Munain Arregui, Javier Ruiz-Martínez, Debora Cerdan, Jacinto Duarte, Jordi Clarimón, Oriol Dols-Icardo, Jon Infante, Juan Marín, Jaime Kulisevsky, Javier Pagonabarraga, Isabel Gonzalez-Aramburu, Antonio Sanchez Rodriguez, María Sierra, Raquel Duran, Clara Ruz, Francisco Vives, Francisco Escamilla-Sevilla, Adolfo Mínguez, Ana Cámara, Yaroslau Compta, Mario Ezquerra, Maria Jose Marti, Manel Fernández, Esteban Muñoz, Rubén Fernández-Santiago, Eduard Tolosa, Francesc Valldeoriola, Pedro García-Ruiz, Maria Jose Gomez Heredia, Francisco Perez Errazquin, Janet Hoenicka, Adriano Jimenez-Escrig, Juan Carlos Martínez-Castrillo, Jose Luis Lopez-Sendon, Irene Martínez Torres, Cesar Tabernero, Lydia Vela, Alexander Zimprich, Lasse Pihlstrom, Sulev Koks, Pille Taba, Kari Majamaa, Ari Siitonen, Njideka U. Okubadejo, Oluwadamilola O. Ojo, UK Brain Expression Consortium (UKBEC), Paola Forabosco, Robert Walker, Kerrin S. Small, Colin Smith, Adaikalavan Ramasamy, John Hardy, Michael E. Weale, Mina Ryten

**Affiliations:** 1 Reta Lila Weston Research Laboratories, Department of Molecular Neuroscience, University College London (UCL) Institute of Neurology, London, UK; 2 Department of Medical & Molecular Genetics, School of Medical Sciences, King’s College London, Guy’s Hospital, London, UK; 3 Departamento de Ingeniería de la Información y las Comunicaciones, Universidad de Murcia, Murcia, Spain; 4 Department of Genetics, King Faisal Specialist Hospital and Research Centre, Riyadh, Saudi Arabia; 5 Lieber Institute for Brain Development, 855 North Wolfe Street, Baltimore, MD USA; 6 Goldsmiths, University of London, New Cross, London, UK; 7 Center for Statistical Genetics, University of Michigan, Ann Arbor, MI USA; 8 Laboratory of Neurogenetics, National Institute on Aging, US National Institutes of Health, Bethesda, MD USA; 9 Data Tecnica International, Glen Echo, MD USA; 10 Department of Twin Research and Genetic Epidemiology, King’s College London, London, UK; 11 Department of Neuropathology, MRC Sudden Death Brain Bank Project, University of Edinburgh, Edinburgh, UK; 12 Singapore Institute for Clinical Sciences, Brenner Centre for Molecular Medicine, Singapore, Singapore; 13 Genomics plc, Oxford, UK; 14 Preventive Neurology Unit, Wolfson Institute of Preventive Medicine, QMUL, London, UK; 15 National Institute of Neurological Disorders and Stroke, Bethesda, MD USA; 16 Department of Computer Science, University of Illinois at Urbana-Champaign, Urbana, IL USA; 17 Department of Molecular Neuroscience, UCL, London, UK; 18 Department of Clinical Neuroscience, University College London, London, UK; 19 Institute of Translational Medicine, University of Liverpool, Liverpool, UK; 20 Biostatistics & Bioinformatics Unit, Institute of Psychological Medicine and Clinical Neuroscience, MRC Centre for Neuropsychiatric Genetics & Genomics, Cardiff, UK; 21 Institute of Healthy Ageing, University College London, London, UK; 22 University of Reading, Reading, UK; 23 MRC Centre for Neuropsychiatric Genetics and Genomics, Cardiff University School of Medicine, Cardiff, UK; 24 UCL Institute of Neurology, London, UK; 25 Institut du Cerveau et de la Moelle épinière, ICM, Inserm U 1127, CNRS, UMR 7225, Sorbonne Universités, UPMC University Paris 06, UMR S 1127, AP-HP, Pitié-Salpêtrière Hospital, Paris, France; 26 Paul Sabatier University, Toulouse, France; 27 Department for Neurodegenerative Diseases, Hertie Institute for Clinical Brain Research, University of Tübingen, Tübingen, Germany; 28 DZNE, German Center for Neurodegenerative Diseases, Tübingen, Germany; 29 Centre for Genetic Epidemiology, Institute for Clinical Epidemiology and Applied Biometry, University of Tubingen, Tübingen, Germany; 30 Departments of Neurology, Neuroscience, and Molecular & Human Genetics, Baylor College of Medicine, Houston, TX USA; 31 Jan and Dan Duncan Neurological Research Institute, Texas Children’s Hospital, Houston, TX USA; 32 Ken and Ruth Davee Department of Neurology, Northwestern University Feinberg School of Medicine, Chicago, IL USA; 33 Northwestern University Feinberg School of Medicine, Chicago, IL USA; 34 Departments of Neurology and Physiology, University of California, San Francisco, CA USA; 35 Gladstone Institute of Neurological Disease; Taube/Koret Center for Neurodegenerative Disease Research, San Francisco, CA USA; 36 National Institutes of Health Division of Clinical Research, NINDS, National Institutes of Health, Bethesda, MD USA; 37 Neurodegenerative Diseases Research Unit, National Institute of Neurological Disorders and Stroke, Bethesda, MD USA; 38 Montreal Neurological Institute and Hospital, Department of Neurology & Neurosurgery, Department of Human Genetics, McGill University, Montréal, QC H3A 0G4 Canada; 39 Department of Human Genetics, McGill University, Montréal, QC H3A 0G4 Canada; 40 Department of Neurology, Leiden University Medical Center, Leiden, Netherlands; 41 Instituto de Biomedicina de Sevilla (IBiS), Hospital Universitario Virgen del Rocío/CSIC/Universidad de Sevilla, Seville, Spain; 42 Fundació Docència i Recerca Mútua de Terrassa and Movement Disorders Unit, Department of Neurology, University Hospital Mutua de Terrassa, Terrassa, Barcelona, Spain; 43 Hospital Universitario Central de Asturias, Oviedo, Spain; 44 Hospital Universitario Parque Tecnologico de la Salud, Granada, Spain; 45 Instituto de Investigación Sanitaria Biodonostia, San Sebastián, Spain; 46 Hospital General de Segovia, Segovia, Spain; 47 Memory Unit, Department of Neurology, IIB Sant Pau, Hospital de la Santa Creu i Sant Pau, Universitat Autònoma de Barcelona, Barcelona, Spain; 48 Centro de Investigación Biomédica en Red en Enfermedades Neurodegenerativas (CIBERNED), Madrid, Spain; 49 Hospital Universitario Marqués de Valdecilla-IDIVAL, Santander, Spain; 50 University of Cantabria, Santander, Spain; 51 Movement Disorders Unit, Department of Neurology, IIB Sant Pau, Hospital de la Santa Creu i Sant Pau, Universitat Autònoma de Barcelona, Barcelona, Spain; 52 Centro de Investigacion Biomedica, Universidad de Granada, Granada, Spain; 53 Hospital Universitario Virgen de las Nieves, Instituto de Investigación Biosanitaria de Granada, Granada, Spain; 54 Hospital Clinic de Barcelona, Barcelona, Spain; 55 Instituto de Investigación Sanitaria Fundación Jiménez Díaz, Madrid, Spain; 56 Hospital Universitario Virgen de la Victoria, Malaga, Spain; 57 Institut de Recerca Sant Joan de Déu, Barcelona, Spain; 58 Hospital Universitario Ramón y Cajal, Madrid, Spain; 59 Department of Neurology, Instituto de Investigación Sanitaria La Fe, Hospital Universitario y Politécnico La Fe, Valencia, Spain; 60 Hospital General de Segovia, Segovia, Spain; 61 Department of Neurology, Hospital Universitario Fundación Alcorcón, Madrid, Spain; 62 Department of Neurology, Medical University of Vienna, Vienna, Austria; 63 Department of Neurology, Oslo University Hospital, Oslo, Norway; 64 Department of Pathophysiology, University of Tartu, Tartu, Estonia; 65 Department of Reproductive Biology, Estonian University of Life Sciences, Tartu, Estonia; 66 Perron Institute for Neurological and Translational Science, Perth, WA Australia; 67 Department of Neurology and Neurosurgery, University of Tartu, Tartu, Estonia; 68 Institute of Clinical Medicine, Department of Neurology, University of Oulu, Oulu, Finland; 69 Department of Neurology and Medical Research Center, Oulu University Hospital, Oulu, Finland; 70 University of Lago, Lagos State, Nigeria; 71 Istituto di Ricerca Genetica e Biomedica, Cittadella Universitaria di Cagliari, 09042 Monserrato, Sardinia Italy

**Keywords:** Gene regulation, RNA splicing, RNA sequencing, Genetics of the nervous system

## Abstract

Genome-wide association studies have generated an increasing number of common genetic variants associated with neurological and psychiatric disease risk. An improved understanding of the genetic control of gene expression in human brain is vital considering this is the likely modus operandum for many causal variants. However, human brain sampling complexities limit the explanatory power of brain-related expression quantitative trait loci (eQTL) and allele-specific expression (ASE) signals. We address this, using paired genomic and transcriptomic data from putamen and substantia nigra from 117 human brains, interrogating regulation at different RNA processing stages and uncovering novel transcripts. We identify disease-relevant regulatory loci, find that splicing eQTLs are enriched for regulatory information of neuron-specific genes, that ASEs provide cell-specific regulatory information with evidence for cellular specificity, and that incomplete annotation of the brain transcriptome limits interpretation of risk loci for neuropsychiatric disease. This resource of regulatory data is accessible through our web server, http://braineacv2.inf.um.es/.

## Abstract (web summary)

Regulation of gene expression and splicing are thought to be tissue-specific. Here, the authors obtain genomic and transcriptomic data from putamen and substantia nigra of 117 neurologically healthy human brains and find that splicing eQTLs are enriched for neuron-specific regulatory information.

## Introduction

The use of genome-wide genotyping in large patient and control populations has resulted in the identification of increasing numbers of common variants that impact on the risk of a wide range of neurological and psychiatric conditions, including Parkinson’s disease^1–3^, Alzheimer’s disease^4–6^, and schizophrenia^7,8^. However, the majority of these risk loci are still poorly characterised, and we do not yet fully understand the underlying molecular and cellular processes through which they act. As it is reasonable to assume that many causal variants operate by regulating gene expression, several studies have attempted to address this problem through the use of expression quantitative trait loci (eQTL) and allele-specific expression (ASE) analyses in a wide range of human tissues, with the aim of finding eQTL and ASE signals that colocalise with disease risk signals^9,10^.

This approach has had success, but perhaps not as much as might have been expected for all diseases. Although the identification of eQTLs in blood has provided insights into autoimmune disorders^11,12^, the utility of brain-related eQTL and ASE data sets, particularly with regard to neurodegenerative disorders, has been harder to demonstrate. For example, monocyte eQTL data sets appear to provide greater insights for Alzheimer’s disease^13^, probably because they reflect regulatory processes in microglia. This would suggest that while relevant eQTL and ASE signals are present in the brain, they are currently difficult to detect given the constraints on eQTL and ASE analyses in human brain.

At present, the most easily available sampling method for brain tissue is post mortem, making repeat sampling impossible and typically leading to smaller sample sizes, particularly for smaller structures such as the substantia nigra. Furthermore, the brain is a highly complex organ. Not only is it split into many regions with known inter-regional differences in expression^14^, each region is composed of an assemblage of different cell types, which complicates the interpretation of transcriptomic data and limits statistical power. Finally, the brain transcriptome is unusual in having a high degree of alternative splicing and a high degree of non-coding RNA activity^15^, much of which has yet to be fully characterised^16^.

We address the latter of these constraints by conducting total RNA sequencing in two basal ganglia regions of clinical interest to human neurodegenerative and neuropsychiatric disorders: the substantia nigra and putamen. Using a comprehensive set of analyses to interrogate different stages of RNA processing and uncover novel unannotated transcripts, we seek to identify not only disease-relevant regulatory loci but also the types of analyses and regulatory positions yielding the most brain and disease-specific information. We find that splicing eQTLs are enriched for neuron-specific regulatory information; that ASE analyses, probably by more effectively controlling for cellular heterogeneity, provide highly cell-specific regulatory information; and that incomplete annotation of the brain transcriptome is limiting the interpretation of risk loci for neuropsychiatric disease. We release the rich resource of eQTL and ASE data generated in this study through a searchable web server, http://braineacv2.inf.um.es/ (Supplementary Fig. 1).

## Results

### RNA quantification and eQTL discovery

**Fig. 1:**
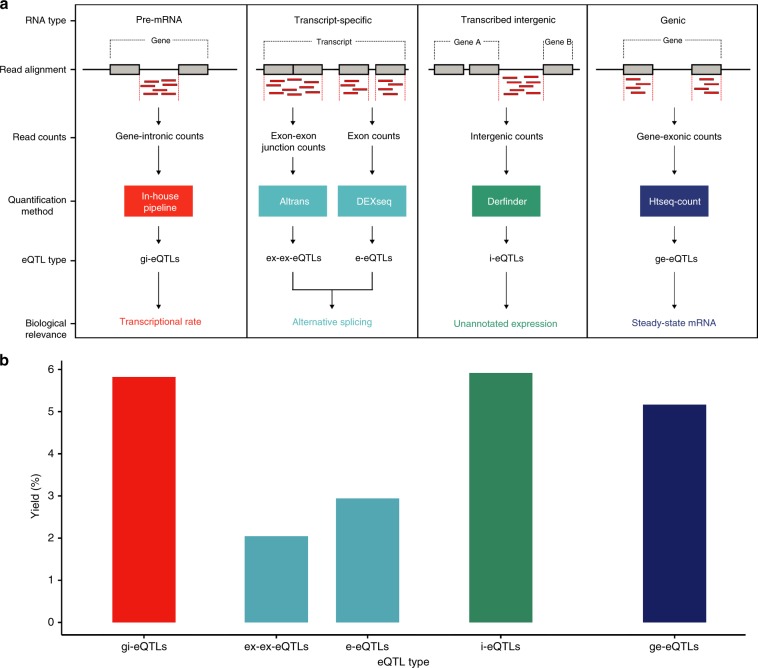
Similar eQTL yield for unannotated expression features compared with annotated features. **a** Overview of transcriptome quantification. RNA was quantified using five pipelines, each targeting distinct stages of RNA processing, and each followed by eQTL generation. Within annotated regions of the transcriptome, reads were mapped to expression features and thereafter RNA was quantified. These features included: all intronic and exonic regions of a gene (producing gene-intronic gi-eQTLs and gene-exonic ge-eQTLs, respectively); individual exons (producing e-eQTLs); and exon–exon junctions (producing ex-ex-eQTLs). As total RNA was used for library construction, reads mapping to introns were presumed to be owing to pre-mRNA within samples (an assumption supported by previous analyses using a subset of these data^69^). Quantification of individual exons and exon–exon junctions provided a means of identifying loci that impact on alternative splicing. In common with most eQTL analyses, we also calculated overall gene expression using all reads mapping to exons of a given gene, resulting in an expression metric that is influenced by transcriptional rate, splicing and RNA degradation rates. Finally, we included annotation-independent approaches to quantify transcription. We focused specifically on unannotated transcription within intergenic regions (producing i-eQTLs, Online Methods). **b** eQTL yields for both tissues were calculated as the number of expression features within a category with at least one significantly associated eQTL divided by the total number of tested features within the same category. Source data are provided as a Source Data file.

We assayed DNA and RNA from 180 brain samples originating from 117 individuals of European descent, which were part of the UK Brain Expression Consortium data set^17^ and which were classified as neurologically healthy based on the absence of neurological disease during life and neuropathological assessment (Supplementary Data 1). We focused on putamen and substantia nigra samples owing to their distinctive expression profiles and disease relevance^18,19^. Using these paired data, we searched for eQTL associations between ~6.5 million genetic variants and ~411,000 RNA expression traits in putamen and ~370,000 RNA expression traits in substantia nigra, resulting in ~5.3 billion eQTL tests. We generated RNA expression traits using both annotation-based (known transcripts) and annotation-agnostic approaches, noting that both types of traits distinguished between the brain regions (Fig. 1a, Supplementary Fig. 2). Within annotated regions, RNA quantification was performed with RNA processing in mind (Fig. 1a) to produce four separate measures of transcription, which formed the bases of our eQTL analysis. This resulted in the generation of four types of eQTLs (gi-eQTLs, e-eQTLs, ex-ex-eQTLs, and ge-eQTLs), of which two were designed to capture the genetic regulation of splicing eQTLs (e-eQTLs and ex-ex-eQTLs). Finally, we included annotation-independent approaches to quantify transcription. We focused specifically on unannotated transcription within intergenic regions (producing i-eQTLs, Methods).

Following stepwise conditional analyses under a false discovery rate (FDR) of 5%, we identified 19,156 separate significant eQTL signals (hereafter, eQTLs) genome-wide (Supplementary Data 2–6), of which 359 were secondary eQTLs (i.e., eQTLs with independent effects after conditioning on the primary eQTL in the region). Although there was a substantial difference in the number of eQTLs identified in putamen and substantia nigra, the difference in sample size (*N* = 111 for putamen and *N* = 69 for substantia nigra) most likely accounted for this. However, eQTL discovery was not simply driven by the number of features tested. Notably, the rate of eQTL discovery, defined as the percentage of all expression features tested with at least one significant associated eQTL, was highest in unannotated intronic and intergenic regions (Fig. 1b), suggesting that such eQTLs could be biologically important.

### eQTLs and i-eQTL target regions show high replication rates

We found that 50.6 and 50.4% of the testable eQTLs identified in putamen and substantia nigra, respectively, were also detected using microarray data, generated by the UK Brain Expression Consortium^17^ and based on a common set of RNA samples. We also found that 39.3% of putamen and 50.6% of substantia nigra eQTLs replicated in the GTEx (v7) data resource^19^, using their 111 putamen and 80 substantia nigra samples respectively. Furthermore, we investigated eQTL replication across all brain regions studied in GTEx (Supplementary Table 1). We demonstrated a replication rate of 19 to 53.3% for putamen, with the highest replication rates observed in cerebellum (53.3%) and caudate (48.2%). In the case of substantia nigra samples, replication rates were more similar across all brain tissues (50.6–62.0%), potentially reflecting the relatively low sample numbers used in the eQTL analysis in both our study (*N* = 65) and that performed by GTEx (*N* = 80). Furthermore, we investigated replication using eQTLs from the larger dorsolateral prefrontal cortex data sets generated by the PsychENCODE and CommonMind consortia^20,21^. We found that although the eQTL data sets generated by PsychENCODE and CommonMind consortia were based on the analysis of sample sets, which are ~13 and ~5 times greater in size than GTEx, the replication rates were (64.2% and 54.2%, respectively, as compared with 52.7% when using the basal ganglia GTEx tissues). In contrast, when we checked our eQTL signals against those reported by Lappalainen and colleagues^10^ using RNA-seq analysis of 373 lymphoblastoid cell lines, we found that despite the larger sample size in this study, only 22.0% of putamen and 24.2% of substantia nigra eQTLs were replicated.

Given the paucity of existing eQTL analyses using annotation-independent approaches, we focused on validating the expression of unannotated intergenic regions that were the target of a significant i-eQTL. Using data provided by GTEx and processed for re-use by recount2^22^, we found that 70.3% of all such i-eQTL target regions (in putamen and substantia nigra combined) were detected in at least one other human tissue within the GTEx data set, with the highest validation rates observed among brain regions (Supplementary Table 1). We also explored the possibility that the transcribed regions detected in our analysis and regulated by i-eQTLs may represent enhancer RNAs as another means of understanding the biological relevance of our findings. Considering all transcribed regions targeted by an i-eQTL and accounting for their genomic size, we found that there was a 3.0-fold increase in overlap with enhancer regions as defined within the GeneHancer database v4.4^23^ among i-eQTL target regions (17.9%) as compared with e-eQTL targets (5.9%), suggesting that the transcribed regions targeted by i-eQTLs are highly enriched for eRNAs.

**Fig. 2:**
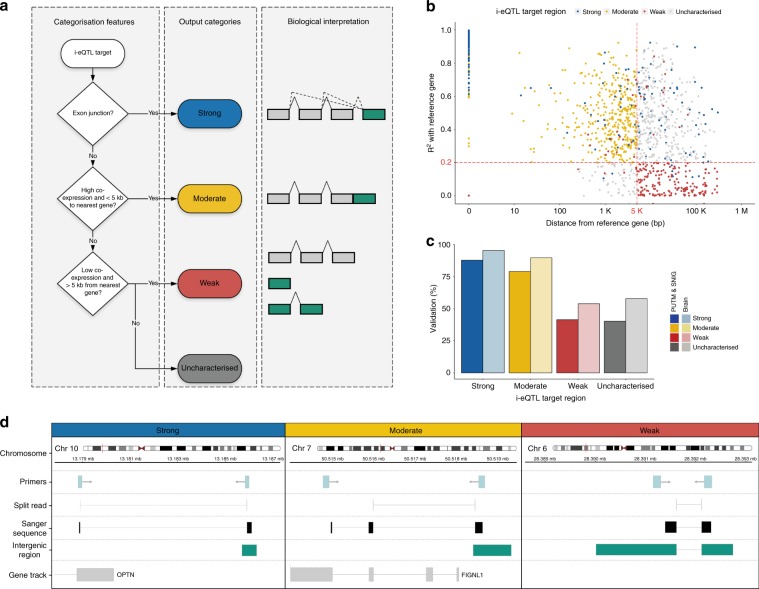
i-eQTL target regions show high replication in independent data sets and validate experimentally. **a** Characterisation of i-eQTL target regions (unannotated expressed regions that were the target of a significant i-eQTL) was based on several features reflecting their relationship to known genes. These features were used to classify these regions into those with strong, moderate, and weak evidence for being part of a known gene. Regions categorised as strong and moderate are considered likely to be novel exons of known genes or misannotations of existing exon boundaries, whereas weak regions are presumed to be independent of any known genes. **b** Scatterplot of genomic distance and correlation of expression between i-eQTL target regions and their reference genes. **c** The expression of unannotated expressed regions was validated in GTEx data, using brain region-specific and global brain expression data. Validation rates in putamen and substantia nigra GTEx expression data were combined and displayed separately from validation rates in RNA-seq data from all GTEx brain regions. **d** Sequencing results for i-eQTL target regions with strong, moderate, and weak evidence of being part of a gene. In each case, tracks are provided relating to the location of the primers used to amplify the unannotated expressed region, the RNA-seq split read, the alignment of Sanger-sequenced cDNA, and the predicted boundaries of the unannotated expression region. Source data are provided as a Source Data file.

We further characterised i-eQTL target regions based on their relationship to known genes (Fig. 2a). Using reads spanning known exons and novel regions, physical proximity and correlation in expression, we categorised unannotated expressed regions into those with strong, moderate, or weak evidence for being part of a known gene (Fig. 2a, Online Methods). This approach allowed us to characterise 68.1% of all unannotated expression regions (Fig. 2b). The validation rate for expression in the GTEx data resource was 98.5% for unannotated expressed regions with strong evidence for being part of a known gene, 93.8% for those with moderate evidence, and still high at 60.4% for regions with weak evidence for being part of a known gene (Fig. 2c). We also selected eight unannotated expressed regions for experimental validation (Supplementary Table 2). Regardless of their putative relationship to existing genes, all eight regions validated using Sanger sequencing (Fig. 2d). In the case of unannotated expressed regions with moderate evidence for association, this analysis also enabled us to clarify the exon boundaries. For example, sequencing confirmed the existence of a novel exon of *FIGNL1* (DER18381, Fig. 2d). Thus, using a combination of public data resources and experimental work, we demonstrated the validity of annotation-independent approaches in transcriptomic analysis.

### i-eQTLs and non-standard eQTLs are largely novel signals

**Fig. 3:**
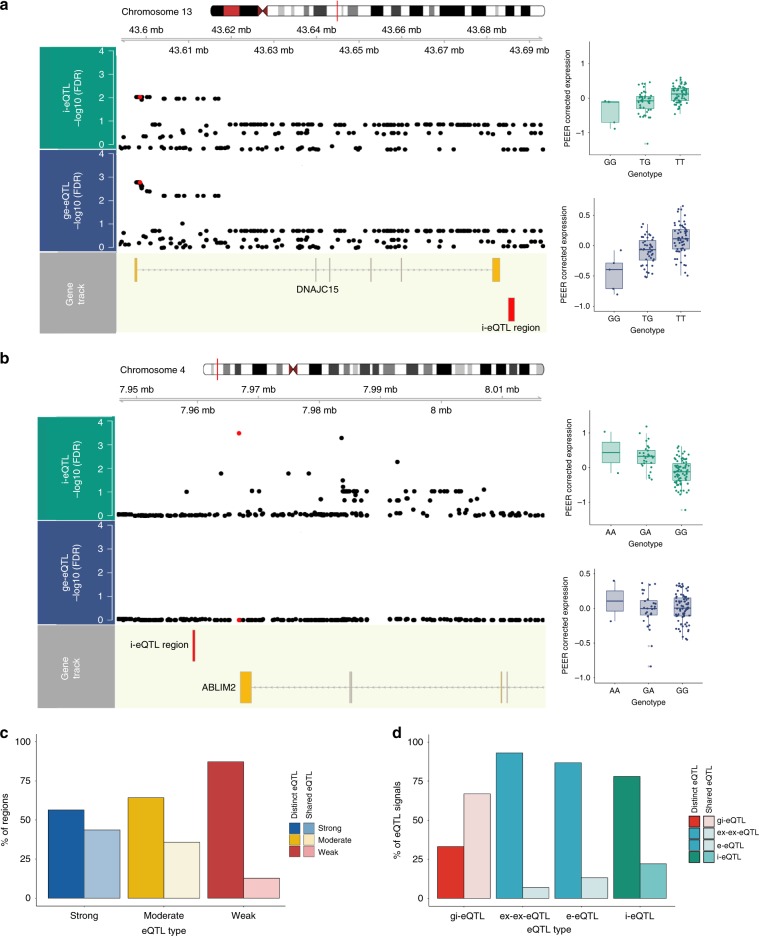
i-eQTL target regions have evidence for distinct regulation. **a** Local association plots (−log10 FDR-corrected *p* values for eQTL association), illustrating sharing of the rs113317084 variant (red point) between the i-eQTL-targeted region, DER32583 (green track), and the ge-eQTL-targeted gene, *DNAJC15* (blue track). **b** Local association plot illustrating no sharing of the rs4696709 variant (red point) between the i-eQTL-targeted region, DER10633 (green track), and the ge-eQTL-targeted gene, *ABLIM2* (blue track). The detection of reads spanning DER10633 and an annotated exon within *ABLIM2* provides compelling evidence that this region represents a novel exon of the gene. **c** Heterogeneity (distinct vs. shared) of i-eQTL signals, cross-categorised by the strength of evidence linking their target region to a known gene, suggests that most are distinct and likely represent novel regulatory variants acting in a transcript-specific manner. Heterogeneity was determined using a modified beta-heterogeneity test, accounting for the dependency structure arising from within-individual and within-gene correlations. i-eQTL beta-coefficients were compared with that of the known exon with most evidence of association with the i-eQTL target region. All eQTL signals with an FDR-corrected *p* value for heterogeneity < 0.05 were considered distinct, whereas those with an FDR-corrected *p* value > 0.05 were considered shared (similar beta-coefficients). **d** Heterogeneity (distinct vs. shared) of non-standard eQTL classes (gi-eQTLs, e-eQTLs, ex-ex-eQTLs, and i-eQTLs) suggests that many of these classes are distinctly regulated. Heterogeneity was determined using a modified beta-heterogeneity test comparing beta-coefficients from ge-eQTLs to those derived from non-standard eQTL analyses applied to the same gene. This analysis was performed separately for gi-eQTLs (tagging pre-mRNA), e-eQTLs, and ex-ex-eQTLs (tagging splicing) and all i-eQTLs (tagging unannotated expression). All eQTL signals with an FDR-corrected *p* value < 0.05 were considered distinct, whereas an FDR-corrected *p* value > 0.05 was taken as evidence of eQTL sharing. Source data are provided as a Source Data file.

As 51.2% of our characterised i-eQTL target regions have strong or moderate evidence linking them to a known gene, we further classified i-eQTLs into those with evidence for being new regulatory variants versus those appearing to act in a consistent manner across all exons (thus recapitulating gene-level signals). Using a modified test of heterogeneity (Methods) we separately analysed i-eQTLs with strong, moderate, and weak evidence for being linked to a known gene (Fig. 3). This analysis demonstrated that some i-eQTLs were indeed re-discovered versions of existing eQTL signals (Fig. 3a). However, many i-eQTLs appear to be independent regulatory sites. For example, SNP rs4696709 regulates DER10633 expression, a probable novel exon of *ABLIM2* (based on the presence of junction reads), but there is no significant co-regulation of other exons of *ABLIM2* (Fig. 3b). Even among those i-eQTLs with strong evidence linking them to a known gene, the percentage of i-eQTLs sharing signals with known annotation expression features was only 44% (Fig. 3c). We extended this analysis to determine whether i-eQTLs with strong or moderate evidence linking them to a known gene could nonetheless be found in the larger eQTL data sets provided by CommonMind, PsychENCODE, and BrainSeq^20,21,24^. In fact, the majority of i-eQTLs were not identified (CommonMind, 73.0%; PsychENCODE, 82.1%, and BrainSeq, 70.0%) again suggesting that the regulatory information captured by these eQTLs is indeed novel. Thus, across all types of characterised i-eQTLs, we found evidence for the majority representing novel regulatory variants, acting in a transcript-specific manner.

We also asked whether our alternative annotation-based eQTL classes (gi-eQTLs, e-eQTLs, and ex-ex-eQTLs) provided novel regulatory information compared with the standard gene-level eQTL analysis (ge-eQTLs). Again we used a modified test of beta-heterogeneity to determine eQTL signal sharing among these classes. Although 66.8% of gi-eQTLs were detectable with standard ge-eQTLs, this figure was only 6.9% for splicing eQTLs (Fig. 3d, suggesting that our additional eQTL classes provided distinct regulatory information driven by splicing effects.

### Splicing eQTLs are enriched for neuronal information

**Fig. 4:**
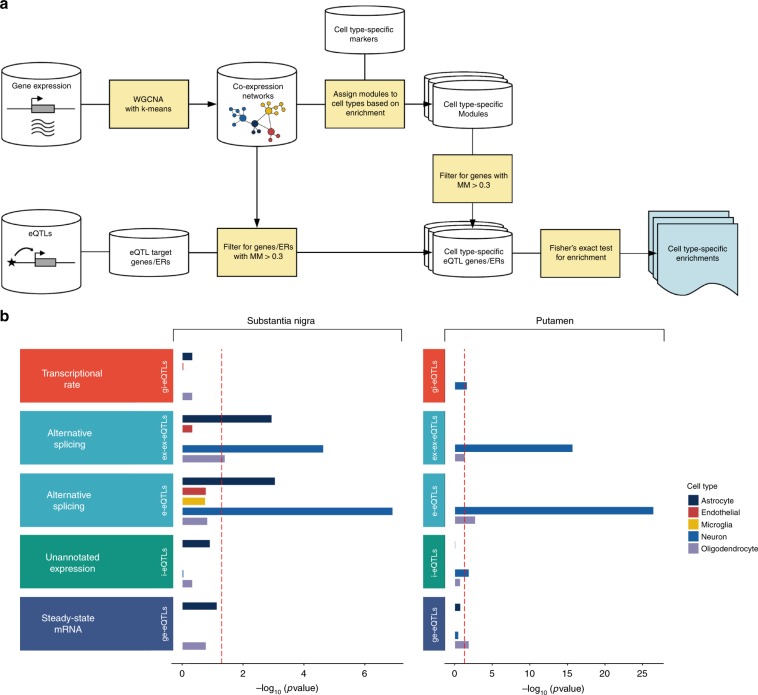
Non-standard eQTL analyses produce additional biologically relevant information. **a** Schematic diagram showing the use of gene co-expression networks to assign eQTL target genes and unannotated expressed regions (ERs) to the cell type most likely to be driving gene expression in the tissue. We used the WGCNA R package^63^. **b** eQTL classes were variably enriched for genes with cell-biased expression, highlighting the importance of capturing this information. Enrichment of genes with cell-biased expression within eQTL targeted expression features was performed separately for each tissue and was determined using a Fisher’s Exact test and a significance cutoff of *P* < 0.05 (dashed red line at −log_10_(*P*) = 1.30). Genes assigned to modules significantly enriched for brain-related cell type markers and with a module membership of > 0.3 were allocated a cell type. Next, for each eQTL targeting a known genic region or an unannotated expressed region with high or moderate evidence linking it to a known gene, if the target gene was allocated to a cell type then the related eQTL received the same cell type label. For eQTLs targeting unannotated expressed regions with low evidence for association with a known gene or which could not be classified, we assigned the target expression feature to a module (and by inference a cell type) based on its highest module membership providing the module membership was at least 0.3. Finally, for each eQTL class and each cell type, namely neuron, microglia, astrocyte, oligodendrocyte, and endothelial cell, we applied a Fisher’s Exact test to test for enrichment of that cell type label among the genes associated to the eQTL class. Expression features targeted by different eQTL classes were variably enriched for genes with cell-biased expression, highlighting the importance of capturing this information. Enrichment of genes with cell-biased expression within eQTL targeted expression features was performed separately for each tissue and was determined using a Fisher’s Exact test and a significance cutoff of *P* < 0.05 (dashed red line at −log_10_(*P*) = 1.30). Source data are provided as a Source Data file.

We asked whether our different eQTL classes varied in terms of the cellular specificity of their target expression features. We used weighted gene co-expression network analysis, in combination with publicly available cell-specific annotation data, to assign eQTL target expression features to one of five broad cell types: neuron, oligodendrocyte, astrocyte, microglia, and endothelial cell (Fig. 4a, Online Methods). This module membership approach allowed us to provide putative cellular classifications for expression features even if they were outside known annotations. We confidently assigned up to 75% of all analysed genes to a specific cell type, and these were then related to 41.5% of all eQTL target expression features. We observed a significant enrichment of neuronal genes in all non-standard eQTL classes in one or both tissues investigated (Fig. 4b, Supplementary Table 2). These included i-eQTLs targeting unannotated expressed regions (FDR-corrected Fisher’s Exact *p* value = 1.20 × 10^−2^ in putamen, Fig. 4b). Furthermore, we found that the targets of splicing eQTLs were significantly enriched for neuronal genes (FDR-corrected Fisher’s Exact *p* values = 1.21 × 10^−7^ and 2.28 × 10^−5^ for e-eQTLs and ex-ex-eQTLs, respectively, in substantia nigra), oligodendrocyte genes (FDR-corrected Fisher’s Exact *p* value = 1.7 × 10^−3^ and 4.1 × 10^−2^ for e-eQTLs and ex-ex-eQTLs, respectively in substantia nigra) and astrocyte genes (FDR-corrected Fisher’s Exact *p* value = 8.74 × 10^−4^ and 1.12 × 10^−3^ for e-eQTLs and ex-ex-eQTLs respectively in substantia nigra, Fig. 4b). This points to the importance of capturing splicing information in the analysis of human brain samples.

### i-eQTLs are enriched for disease-relevant information

We investigated the overlap of unannotated transcribed regions and eQTL sites with known GWAS association signals. We used the US National Human Genome Research Institute/European Bioinformatics Institute (NHGRI-EBI) GWAS catalogue, restricted to genome-wide significant SNPs (*P* < 5 × 10^−8^) and stratifying for SNP-phenotype associations of relevance to neurological/behavioural disorders as defined within the STOPGAP database^25^. First, we investigated the possibility that unannotated transcribed regions could themselves harbour risk loci of relevance to neurological diseases, by calculating the proportion of transcribed intergenic regions containing a brain-relevant risk locus and comparing this value to that for expressed exons. After adjusting for the size of each annotation, we found that unannotated transcribed regions and exons had a similar level of overlap with brain-relevant risk loci (3.5% for exons and 2.9% for transcribed intergenic regions after adjustment for annotation size). However, the enrichment of brain-relevant risk loci was higher for novel transcribed regions as compared to exons (1.51-fold for targets of e-eQTLs versus 2.70 fold for targets of i-eQTLs after adjustment for annotation size). Furthermore, we found a significant enrichment for GWAS variants that were associated with neurological and behavioural disorders compared with all other SNP-phenotype associations (Supplementary Fig. 3) among our eQTLs. Although the enrichment of brain-relevant GWAS associations was most evident in ex-ex-eQTLs and gi-eQTLs, we also found a significant enrichment for i-eQTLs (FDR-corrected Fisher’s Exact *p* value = 6.45 × 10^−7^). i-eQTLs provided useful information for 36.7% of all the neurologically relevant risk loci within this analysis (equating to 76 loci). Given that these findings could potentially be driven by correlations between i-eQTLs and more conventional eQTL signals, we repeated this analysis only using i-eQTLs considered to be independent regulatory signals (based on modified beta-heterogeneity testing described above). We found that the enrichment of brain-relevant risk loci among i-eQTLs increased in significance in this sub-group (FDR-corrected Fisher’s Exact *p* value = 7.01 × 10^−8^). Thus, our analysis suggests that i-eQTLs do contribute to the understanding of a significant proportion of neurologically relevant risk loci.

**Fig. 5:**
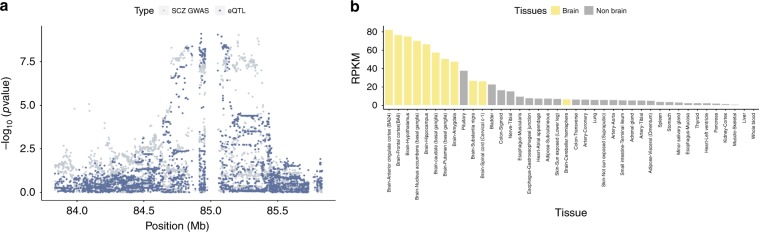
Annotation-independent approaches yield disease-relevant information. **a** Colocalisation of the schizophrenia GWAS lead SNP rs950169 (GWAS *p* value = 7.62 × 10^−11^) and the i-eQTL targeting DER36302 (eQTL *p* value = 1.15 × 10^−10^ in putamen). **b** Expression of DER36302 across tissues sampled by the GTEx consortium. Brain tissues are highlighted in yellow with the anterior cingulate and frontal cortex showing the highest expression. Source data are provided as a Source Data file.

We further explored signal enrichment in i-eQTLs in relation to two neurological diseases related to basal ganglia dysfunction: Parkinson’s disease and schizophrenia. Using GWAS summary statistics for these diseases^1,7^, we performed colocalisation analyses for disease-risk association signals against i-eQTL signals using the *coloc*^26^ programme. We identified 23 i-eQTL signals that colocalised with risk loci for schizophrenia or Parkinson’s disease (Supplementary Data 8). Among the former, we identified a signal indexed by the lead SNP rs35774874 (GWAS *p* value = 1.97 × 10^−11^) that colocalised with an i-eQTL targeting a probable novel 3′-UTR of *SNX19* (posterior colocalisation probability = 0.75), a gene that has already been highlighted in schizophrenia^27,28^. Similarly, we identified a colocalisation of the Parkinson’s disease GWAS lead SNP rs4566208 (GWAS *p* value = 2.28 × 10^−7^) with an i-eQTL regulating a probable novel exon of *ZSWIM7* (i-eQTL *p* value = 1.09 × 10^−5^; posterior colocalisation probability = 0.89, Supplementary Fig. 4). However, we also found seven co-localising i-eQTL signals targeting unannotated expressed regions that were not linked to a known gene. For example, we found that the schizophrenia risk SNP rs12908161 (GWAS *p* value = 9.41 × 10^−10^) had a posterior colocalisation probability of 1.00 with an i-eQTL targeting the unannotated expressed region DER36302 (chr15:84833811-84833975, eQTL *p* value = 7.94 × 10^−10^ in putamen, Fig. 5a). This novel transcribed region appears to be independent of neighbouring genes, and is expressed in human brain, with the highest expression in the anterior cingulate and frontal cortex, two brain regions relevant to schizophrenia (Fig. 5b).

### ASE discovery and validation

**Fig. 6:**
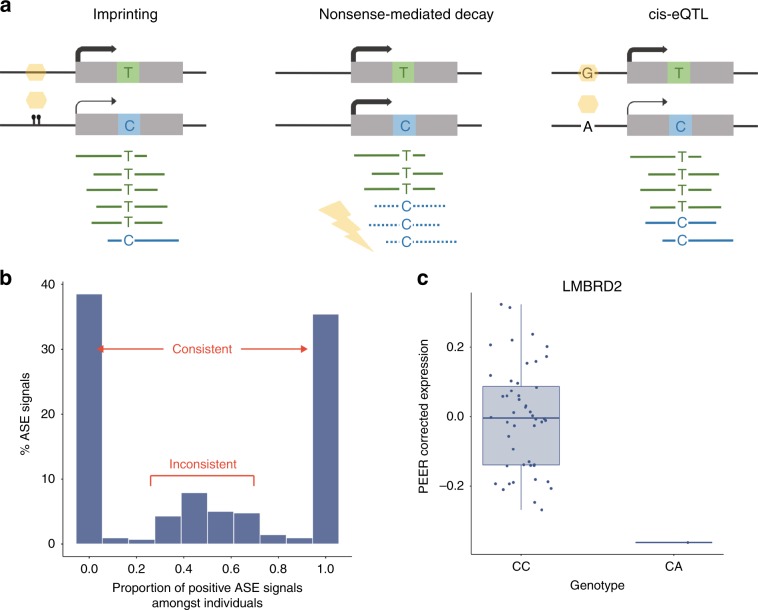
Allele-specific expression provides evidence of dosage compensation. **a** Overview of the mechanisms by which allele-specific expression can arise. Allele-specific expression can arise through epigenetic effects (e.g., imprinting), heterozygous mutations triggering nonsense-mediated decay of transcripts, and regulation by (for example) a cis-regulatory variant (cis-eQTL). **b** The majority of allele-specific expression signals passing FDR < 0.05 produced unidirectional signals (0 or 1) and were considered consistent. Inconsistent ASE signals (those that were not unidirectional in ≥ 10 individuals) were found only in known imprinted genes, thus providing additional validation of our ASE signals. **c** Comparison of *LMBRD2* expression in putamen from one individual heterozygous for a rare stop gain mutation (CA) in the gene versus all other individuals (CC) revealed a significant reduction in *LMBRD2* expression, implying effective nonsense-mediated decay. Data presented using Tukey-style box plots. Source data are provided as a Source Data file.

We applied ASE analysis to a subset of 84 brain samples (substantia nigra *n* = 35; putamen *n* = 49) for which we had access to whole-exome sequencing in addition to SNP genotyping data (Methods, Supplementary Data 1). ASE analysis quantifies the variation in expression between two haplotypes of a diploid individual distinguished by heterozygous genetic variation, and so can capture the effects of a range of regulatory processes, namely genomic imprinting, nonsense-mediated decay and cis-regulation (Fig. 6a). In total 252,742 valid heterozygous SNPs (hetSNPs) across 53 individuals were analysed. Of these, 7.62% (19,266) were significant ASE signals (hereafter, ASEs) at FDR < 5% in at least one sample, covering 8654 genes. Of the 19,266 ASEs identified, 12,096 were found in putamen and 11,871 in substantia nigra (Supplementary Data 9). Consistent with previous studies, we found that ASEs can operate as markers of imprinting or parent-of-origin effects: ASE signals that are not unidirectional across individuals are expected to be enriched for imprinted genes (Fig. 6a). Consistent with expectation, of all genes containing an ASE, 170 were identified on the X chromosome (equating to 1.96%). Furthermore, we observed that all inconsistent ASE signals (those that were not unidirectional within ≥ 10 individuals) were located within genes known to be imprinted (as reported in www.geneimprint.com or within the literature^27–30^) compared with 1–5% of consistent signals (Fig. 6b).

We also found evidence for the generation of ASE signals through nonsense-mediated decay. We identified 61 protein-truncating variants (defined as stop gain, donor splice site, and donor acceptor mutations) among our ASEs. Consistent with expectation, the majority of these variants were predicted to cause nonsense-mediated decay (52.5% using SNPeff^31^) and appeared to result in mono-allelic expression, with >95% of all reads at the ASE site originating from a single allele. These extreme ASEs are expected to generate an effective reduction in gene dosage, and therefore to cause a significant reduction in the total expression of the affected genes. An example of this pattern is seen in the *LMBRD2* gene (Fig. 6c).

Finally, to check the overall reliability of our findings, we looked for validation of our ASEs in an independent data set of 462 lymphoblastoid cell lines reported by Lappalainen and colleagues^10^. We found that 67% of testable ASE signals could be detected at an FDR < 5%, demonstrating the reliability of our ASE sites while also suggesting the presence of brain-specific ASEs.

### ASEs tag both gene level and splicing eQTLs

Cis-regulatory variants are known to be one important generator of ASEs^32^. We therefore investigated the overlap of eQTLs with ASEs in our data, and compared it with the overlap observed with randomly selected non-ASE heterozygous SNPs. After controlling for read depth, we identified a highly significant enrichment of eQTLs among our ASEs (*p* value = 2.65 × 10^−195^ in putamen and 9.99 × 10^−111^ in substantia nigra, using a randomisation approach—see Online Methods). This enrichment remained significant when we restricted our analysis to eQTLs with effects on splicing (e-eQTLs and ex-ex-eQTLs, *p* value = 1.17 × 10^−178^ in putamen and 1.29 × 10^−89^ in substantia nigra, using a randomisation approach). However, as expected, it was absent when we considered ASEs located within imprinted genes, where the parental origin of the SNP rather than the impact of cis-regulatory sites is expected to drive allele-specific expression (*p* value = 0.923 in putamen and 0.856 in substantia nigra, using a randomisation approach).

To investigate the extent to which ASE sites tagged gene-level or transcript-specific cis-regulatory effects, we focussed on common ASE sites (seen in ≥10 individuals) that were unidirectional in nature (same direction of effect across all individuals). For each valid ASE site, we measured exon expression across all three genotypes. Of all testable ASEs, we found that 43.2% were also likely to be eQTLs. To ask whether the underlying cis-regulatory effects operated in an exon-specific or gene-level manner, we repeated the analysis using gene-level expression across the genotypes. Of the ASEs that were also likely eQTLs, 51.8% appeared to operate in an exon-specific manner, implying that they tagged splicing eQTLs. rs7724759, a splice site variant present in the *CAST* gene (Supplementary Fig. 6), and rs1050078, a variant in *SNX19* (Supplementary Fig. 6), are examples of ASEs likely to be driven by exon-level and gene-level regulation respectively.

Although this approach allowed us to identify ASE sites tagging splicing eQTLs, it was limited to a small subset of common ASE sites and represented only 0.8% of all ASEs. To address this issue, we used the machine learning programme SPIDEX to predict the effect of all ASEs on splicing^33^. Given a genetic variant, SPIDEX provides the delta percent inclusion ratio (ΔΨ) for the exon in which the variant is located (reported as the maximum ΔΨ across tissues). We compared predicted ΔΨ values at ASE sites versus non-ASE sites and found significantly higher values among ASEs (Fisher’s Exact test *p* value = 4.50 × 10^−5^ and *p* value = 2.07 × 10^−19^ using a randomisation approach). This strongly suggests that ASEs are enriched for variants with effects on splicing.

### ASEs show biologically and disease-relevant enrichments

**Fig. 7:**
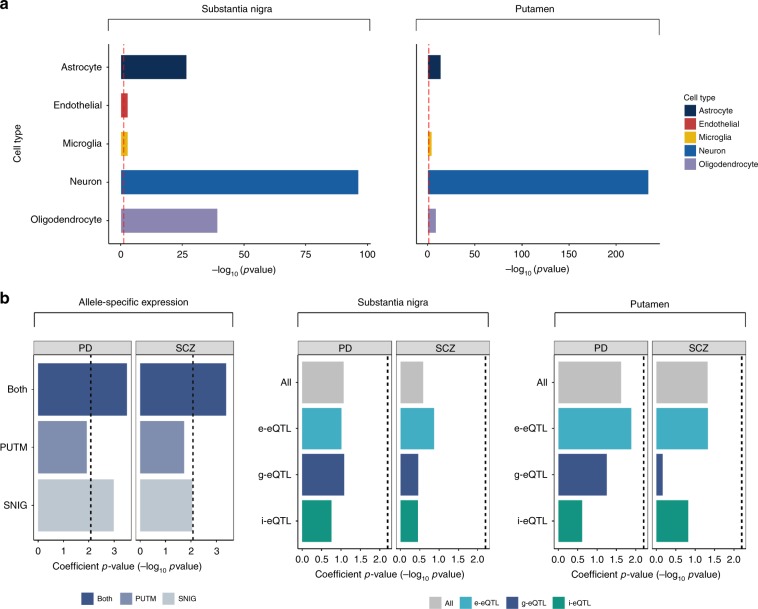
Allele-specific expression sites biologically and disease-relevant information. **a** Genic locations of ASEs were highly enriched for genes with cell-biased expression in both putamen and substantia nigra. Enrichment of genes with cell-biased expression within ASE locations was performed separately for each tissue and was determined using a Fisher’s Exact test and a significance cutoff of *p* value < 0.05 (dashed red line at −log_10_(*P*) = 1.30). **b** Enrichment of heritability for Parkinson’s Disease and schizophrenia in ASEs and eQTLs identified in substantia nigra, putamen and across both tissues. Source data are provided as a Source Data file.

We assessed the cellular specificity of the regulatory information ASEs provide. Given that ASE analysis is performed within an individual and so is not subject to the confounding effects of cellular heterogeneity across individuals, we expected that ASEs would be a powerful means of obtaining cell-specific regulatory information. Using a similar approach to that applied to eQTLs to assign genes containing ASEs to brain-relevant cell types (neurons, oligodendrocytes, astrocytes, microglia, and endothelial cells), we found that ASE-containing genes were highly enriched for neuronally expressed genes (Fisher’s Exact test FDR-corrected *p* values of 9.97 × 10^−235^ in putamen and 3.05 × 10^−97^ in substantia nigra). We also found significant enrichments (FDR *p* value < 5%) for oligodendrocyte, astrocyte, microglia, and endothelial gene sets. Although we observed a similar pattern of cell type-specific enrichments among eQTLs, the strength of evidence for cellular specificity of ASEs was striking, suggesting that incomplete covariate correction may be hampering the power of eQTL analyses (Fig. 7a, Supplementary Data 7).

Finally, we used GWAS summary data sets for Parkinson’s disease^1^ and schizophrenia^7^ to investigate the disease relevance of ASEs. As GWAS loci often lie close to genic regions and so are likely to overlap by chance with ASE signals, we used a randomisation approach^34^ to investigate the enrichment of GWAS loci within our ASEs (Methods). We compared overlaps between risk loci and ASEs to overlaps between risk loci and randomly selected non-ASE sites, and found a highly significant enrichment of GWAS risk loci for both schizophrenia (*p* value = 7.49 × 10^−35^, using a randomisation approach, for ASEs derived from both tissues) and Parkinson’s disease (*p* value = 4.19 × 10^−7^, using a randomisation approach, for ASEs derived from both tissues). We validated these findings using stratified LD score regression^35^ by treating our ASE sites as a form of binary annotation, and interestingly found that the enrichment of Parkinson’s disease heritability appeared to be more specific to ASEs identified in substantia nigra using this approach (Fig. 7b, Supplementary Data 10). There was no enrichment in Parkinson’s disease or schizophrenia heritability among eQTLs using the same method. Thus, although we recognise that eQTLs can be powerful when linked to even more specific cell types for this type of analysis^20,36^, we demonstrate the additional power of ASE analysis to generate disease-relevant information, despite the small number of samples we had at our disposal.

## Discussion

The human brain is an especially challenging organ in which to conduct eQTL and ASE studies. In addition to the difficulties of sample collection, the brain is a highly complex organ, with site-specific pathologies that motivate the use of equivalently specific analyses. The brain is also known to express many transcripts not seen in other parts of the body, and it is suspected that much of its transcriptome remains uncharacterised^16,37^.

We tackled these issues by collecting RNA-seq data from human substantia nigra and putamen, and applied a bank of five transcriptome quantification methods, including annotation-agnostic approaches as well as approaches to interrogate different stages of RNA processing. We found that there is significant variation among eQTL classes in their neuron- and brain-specific information content, as measured by the cell type-specific enrichment of eQTL targets. The most neuronally enriched and brain-specific results were found in eQTL classes that most closely tag the regulation of splicing (e-eQTLs and ex-ex-eQTLs) rather than gene-level expression. This finding is consistent with recent studies that suggest that splicing eQTLs can provide significant insights into complex diseases in general^38^, and brain-related disorders in particular (e.g., schizophrenia^20,36^). Thus, in addition to providing a rich eQTL resource, our study suggests that the utility of existing and future eQTL analyses in human brain may critically depend on the ability of the RNA sequencing technology, and of the analytic methods applied, to capture transcript-specific information.

We also asked whether the incomplete annotation of the human brain transcriptome might be limiting eQTL discovery as well as reducing the tissue-specific nature of the regulatory information discovered. We focused on transcription within intergenic regions, as transcriptional activity in these regions cannot be explained by the presence of pre-mRNA but instead could be generated through the expression of long intergenic non-coding RNAs and enhancer RNAs, which are reported to be expressed in a highly tissue-specific manner^15,39^. We show that these expressed intergenic regions are reliably detected, and that ~16.1% of these expressed regions are highly likely to represent novel exons of known genes (as demonstrated through the existence of junction spanning reads). They are also enriched for overlap with enhancer regions, suggesting that many could also represent eRNAs (3.03-fold enrichment over expressed exons). Finally, we show that intergenic eQTLs (i-eQTLs) are enriched for neuronally relevant information, and most importantly that they can provide unique disease insights that would be missed using standard analyses, as illustrated by the colocalisation of i-eQTL signals with schizophrenia risk loci.

Nevertheless, the identification of splicing eQTLs from homogenates of macro-dissected human brain, particularly from brain regions that are hard to obtain in large numbers, is likely to remain challenging even after accounting for the on-going development of tools to optimise transcriptome quantification. This motivates the use of ASE analysis, a form of within-individual comparison that compares variation in expression between two haplotypes of a diploid individual. This within-individual comparison means that ASE analysis is unaffected by between-individual confounders, such as the variability in cell type-specific density among individuals. We applied ASE analysis to 49 putamen and 35 substantia nigra samples, for which both whole-exome sequencing and genotyping data were available. Consistent with our expectation, we found that ASEs were significantly enriched for variants identified as splicing eQTLs within our own analysis or predicted to affect splicing according to SPIDEX. Furthermore, we found that the ASEs we identified tagged regulatory information that was highly enriched for neurons and brain-relevant cell types, even after accounting for the general enrichment in brain-specific information contained within the RNA-seq data. Finally, and most importantly, we used two separate approaches to demonstrate the relevance of ASEs to both Parkinson’s disease and schizophrenia, with evidence for enriched heritability among ASEs. Given the small numbers of samples used in our ASE analyses, this finding is particularly striking. Thus, we provide evidence to suggest that ASE analysis may be a particularly effective and efficient means of obtaining regulatory information relevant to splicing, cell type and disease.

In summary, by using a range of methods to quantify and analyse brain transcriptomic data, we demonstrate the importance of capturing information on the regulation of known and novel splicing for the understanding of complex brain disorders, and show that ASE analyses performed even on small sample sets can provide additional insights.

## Methods

### Generation and processing of RNA sequencing data

Human brain samples originating from 117 individuals of European descent were obtained from the Medical Research Council (MRC) Sudden Death Brain and Tissue Bank and the Sun Health Research Institute. All samples were authorised for ethically approved scientific investigation (Research Ethics Committee number 10/H0716/3, National Hospital for Neurology and Neurosurgery and Institute of Neurology Research Ethics Committee) and had fully informed consent for retrieval. These samples constituted a subset of the United Kingdom Brain Expression Consortium (UKBEC) data set. RNA was isolated by using the miRNeasy 96 sample kit (Qiagen, UK) and the RNA integrity number was assessed for each sample using the Agilent 2100 Bioanalyzer (Agilent Technologies UK Ltd, UK) in combination with the RNA 6000 Nano-LabChip kit (Supplementary Table 1).

cDNA libraries were prepared by the UK Brain Expression Consortium in conjunction with AROS Applied Biotechnology A/S (Aarhus, Denmark). Using 100 ng of total RNA as input, amplified cDNA was generated with the NuGen Ovation RNA-seq System V2 (NuGen Technologies, US) according to the manufacturer’s protocol. This protocol deselects for rRNA by using oligo dT and random hexamer primers for reverse transcription. After fragmentation of cDNA (1 μg) using a Covaris S220 Ultrasonicator, we used the Illumina TruSeq DNA library preparation kit (Illumina, US) followed by 10 cycles of PCR amplification of library molecules containing adaptor molecules on both ends to generate the final libraries. Sequencing of these DNA libraries was performed with Illumina’s TruSeq V3 chemistry/HiSeq2000 to generate 100 base pair paired-end reads. Illumina’s CASAVA Software was then used to generate fastq-files. Paired-end data were mapped to the human genome (build GRCh37) using tophat2^40^ (v2.0.9) with default settings and a transcriptome-guided approach using the Ensembl reference (v72) based on GENCODE version 18. Reads mapping to rRNA regions were removed from the analysis.

Transcriptome quantification was performed using the transcriptome definition from Ensembl reference (v72) using HTSeq-Counts^41^ (v0.5.4p4) for exonic regions, BEDtools^42^ for intronic regions, DEXSeq^43^ (v1.10.6) for individual exons, Altrans^44^ (v1.1.02) for exon–exon junctions and the derfinder R package^45^ for unannotated transcribed regions. Expressed regions were identified by derfinder using default settings and filtering for expressed regions of >100 bp in length, annotated as intergenic based on Ensembl v72 and UCSC through the R library TxDb.Hsapiens.UCSC.hg19.knownGene version v3.1.2, and which uniquely mapped (defined as >98% alignment precision). This approach was applied to each tissue separately (putamen and substantia nigra). All forms of transcriptome quantification were normalised using CQN^46^, with GC content calculated separately for each type of quantification and used as an input for the CQN R-bioconductor package. The resulting normalised expression data were then transformed into Reads Per Kilobase of transcript per Million (RPKM) values and log2 converted. Exonic, intronic, and transcribed intergenic regions with an RPKM of >0.1 in at least 80% of samples were selected for downstream analyses.

### DNA extraction and genotyping

DNA was extracted from sub-dissected samples (100–200 mg) of human post mortem brain tissue using Qiagen’s DNeasy Blood & Tissue Kit (Qiagen, UK). All samples were genotyped on the Illumina Infinium Omni1-Quad BeadChip and on the Immunochip, a custom genotyping array designed for the fine mapping of autoimmune disorders. Standard quality control on the merged genotyping data was performed. Individuals of suspected non-European descent and samples with percentage of non-missing genotypes of < 95% were removed from the analysis. Reported gender status and non relatedness of samples were confirmed. Monomorphic SNPs, variants with missing position information, variants with a *p* value < 0.0001 for deviation from Hardy–Weinberg equilibrium, variants with a genotype call rate < 95%, variants with less than two heterozygotes present and variants with mismatching alleles from 1000 Genomes Project were removed from the analysis. Imputation was performed using MaCH^47^ and minimac^48^ using the European panel of the 1000 Genomes Project (March 2012: Integrated Phase I haplotype release version 3, based on the 2010 November data freeze and 14 March 2012 haplotypes). We used the resulting 5,878,211 SNPs and 576,942 indels with good postimputation quality (*R*^2^ > 0.50) and minor allele frequency (MAF) of at least 5%.

A subset of DNA samples were also analysed using whole-exome sequencing^49^ (*N* = 57, Supplementary Data 1, EGAS00001002113). Exome sequencing was performed on each subject of the cohort according to the manufacturer’s capture protocol; capture kits included Illumina and Nimblegen. Paired-end sequence reads were aligned with BWA against the reference human genome (UCSC hg19)^50^. Duplicate read removal, format conversion, and indexing was performed with Picard (http://picard.sourceforge.net/). The Genome Analysis Toolkit was used to recalibrate base quality scores, perform local realignments around indels, and to call and filter the variants^51,52^. SnpEff was used to annotate gene and effect information for the variants^31^. Subject QC was performed using typical methods to check for call rate, heterozygosity outliers, gender, relatedness, and population outliers. These QC checks were performed using Plink^53^ based on the Single Nucleotide Variants (SNVs) that are in the intersection of the exome capture kits used. VCFTools (http://vcftools.sourceforge.net/) was used to convert the SNV vcf file to Plink formatted genotypes. Population outlier checks included subjects, populations, and genotypes from the 1000 Genomes Project^54^, Phase1 Release v2.20101123 for reference. Individual genotypes were removed with genotype quality Phred-scores below 40. Omni-1M, Immunochip and exome sequencing data were then merged with priority placed on the genotyping array data, only overwriting calls from arrays when genotyping data were missing. MaCH^47^ was used to determine phasing and the resulting files were converted into a variant call format (VCF) file using R scripts. An average of 286,824 SNPs with heterozygous genotypes were obtained per individual.

### eQTL discovery and replication

Prior to performing eQTL analyses, the Probabilistic Estimation of Expression Residuals^55^ (PEER) method was used to identify unknown factors affecting expression levels and so optimise eQTL discovery. PEER was run using default parameters with brain regions, age, and gender accounted for as known covariates to generate 13 unknown factors (captured through the PEER axes), which were applied to RPKM normalised values to produce residuals. As would be expected, many of the 13 unknown factors were highly correlated with recognised covariates such as RIN, library batch, and intronic read rate (Supplementary Fig. 5). We also identified nine factors that were highly correlated with the specific brain region analysed. We found that expression of *SLC6A3*, which encodes the dopamine transporter (expressed with very high specificity in dopaminergic neurons of the ventral midbrain including the substantia nigra, see below), was highly correlated with the 10th PEER axis (*p* value: 1.9 × 10^−26^).

Variants within ±1 Mb of each normalised expression feature (i.e., gene, exon, exon–exon junction, and unannotated intergenic region) were tested for cis-eQTL discovery using the R package MatrixEQTL^56^. Gender, age, and the first three genetic principal component vectors were included as covariates within the linear model of marker genotype (imputed expected counts of minor allele) against PEER corrected expression values. The Benjamini–Hochberg method was applied to calculate the FDR adjusting for the tests performed for each transcriptomic feature. Finally, we used a stepwise conditional analysis for each quantification type to detect independent variant effects targeting the same expression feature.

Three major types of data were used to validate eQTL results: (i) eQTL data reported by Ramasamy and colleagues^17^, which used an overlapping set of donor samples but a hybridisation-based microarray method for exon-level transcriptome quantification, (ii) eQTL data generated by the GTEx^19^, PsychENCODE and CommonMind Consortia^20,21^, which were based on independent sample sets, but assayed multiple brain regions and which used RNA sequencing to quantify the transcriptome, and (iii) eQTL data relating to 373 lymphoblastoid cell lines generated by the Geuvadis consortium^10^, which used RNA sequencing for transcriptome quantification. Gene-level eQTLs (ge-eQTLs) identified within our study were declared as replicated when the significant SNP-gene pair was declared a hit with an FDR < 5% in both our data set and the comparison data set.

### Characterisation of unannotated transcribed intergenic regions

We leveraged information from split reads (reads aligning to a genomic location with a gap or multiple gaps) and combined this with information on co-expression and physical proximity to known genes to characterise novel transcribed regions. In the first instance, the split read information for each sample was collected from the Tophat2^40^ junction output file, and overlaps between transcribed intergenic regions targeted by eQTLs and split reads were assessed using the GenomicRanges^57^ R package. If a transcribed region overlapped with a split read, then the same split read was screened for overlap with known genes. Only transcribed intergenic regions with split reads present in at least four separate samples were classed as having high evidence for being part of a known gene. In the absence of relevant split read data, the nearest gene (in genomic distance) from each transcribed intergenic region was selected using the GenomicRanges^57^ R package and the *r*² was calculated between the expression of the transcribed intergenic region and each exon of the nearest gene. Transcribed regions that had a maximum *r*² > 0.2 and were <5Kb from the nearest gene were classed as having moderate evidence for being part of a known gene. Transcribed intergenic regions were classed as having low evidence for being part of a known gene when the *r*^2^ was  <0.2 or they were >5Kb from the nearest gene.

We also explored the possibility that unannotated transcribed regions could represent enhancer RNAs (eRNAs). We used the GeneHancer database v4.4^23^, which draws information from a wide range of sources (including ENCODE, the FANTOM5 atlas, the VISTA Enhance Browser, dbSUPER and EPDnew, and UCNEbase) to define enhancer locations and then quantified the percentage overlap between eQTL target regions and enhancer regions. We adjusted for differences in the genomic size of eQTL target regions by calculating the percentage overlap per Mb of transcribed target sequence.

### Validation of unannotated transcribed intergenic regions

We validated the transcription of intergenic regions targeted by i-eQTLs in silico using the data available on tissue-specific transcription generated by the GTEx consortium (www.gtexportal.org) and mapped through recount2^22^. The genome coordinates of all intergenic regions of interest were quantified from the transcription expression profiles generated with derfinder and available in recount2^22^. Counts were transformed to RPKMs and validation of transcription was considered when the region had an RPKM > 0.1 in at least 80% of samples in the tissue of interest.

We selected eight transcribed intergenic regions targeted by i-eQTLs for validation by Sanger sequencing. All regions were detected through analysis of putamen samples and we used a subset of RNA samples from the set of 111 used for RNA sequencing (Supplementary Table 2). In each case, reverse transcription was performed with 500 µg of total RNA using High Capacity cDNA RT Kit (Applied Biosystems) and random primers as per manufacturer’s instructions. PCR was performed using specific primers (Supplementary Table 2), all of which were designed to span predicted exon–exon junctions, and FastStart PCR Master (Roche). Amplification of the predicted band was confirmed by agarose gel electrophoresis and following confirmation, enzymatic clean-up of PCR products was performed using Exonuclease I (Thermo Scientific) and FastAP Thermosensitive Alkaline Phosphatase (Thermo Scientific). Sequencing was performed using the BigDye terminator kit (Applied Biosystems). All sequences were viewed using CodonCode Aligner (V. 6.0.2).

### Identification of redundant eQTLs by beta-hetereogeneity testing

A mixed model approach was used to identify heterogeneity in eQTL signal strength (beta coefficient or slope) within a gene. To test for beta-heterogeneity between ge-eQTL and each other eQTL class (namely gi-eQTLs, e-eQTLs, ex-ex-eQTLs and relevant i-eQTLs) targeting the same gene, two models were fitted: (1) a non-heterogeneous (single slope) model with allele dosage as the main effect and two random effects, one indexing individuals and one indexing genes or exons or exon junctions and (2) a heterogeneous (multiple slope) model containing the same terms, but with the addition of a fixed-effect allele dosage × exon-or-exon junction index interaction term. The lme4 R package was used to fit both models. A *p* value for the likelihood ratio test comparing the two models was generated with the R function anova() and an FDR of <5% was applied to assess significance.

### Identification of disease-relevant eQTLs

We used the STOPGAP database^25^ (accessed on 20th of March 2018) to access and sub-classify loci identified through genome-wide association studies (GWAS). eQTL-GWAS overlap was checked using all eQTLs passing an FDR < 5%. The percentage overlap and enrichment was calculated based on the total number of eQTLs identified. In addition, summary statistics were obtained from Parkinson’s disease (with the exclusion of data generated by 23andMe) and schizophrenia GWAS^1,7^. We applied *coloc*^26^ to colocalise Parkinson’s disease and schizophrenia loci with our eQTL signals. For each locus with a GWAS *p* value of <1 × 10^−5^ we examined all SNPs available in both data sets within 1 Mb of the GWAS SNP of interest, and ran *coloc* with default parameters and priors. We called the signals colocalised when Coloc H3 + H4 probabilities were greater than 0.8 and the H3/H4 probability ratio was greater than 2.

### ASE signal discovery and replication

The ASE discovery pipeline was motivated by the method of Rozowsky and colleagues^58^ and involved the creation of parent haploids using phased variant data to reduce the impact of mapping biases on ASE identification. Each individual’s haploid genome was constructed using genotype data in the relevant VCF file, as parental information was unavailable. Deviations from the reference genome present in the VCF file were used to update the reference and create an artificial genome (two haploid genomes originating from each parent) using the Personal Genome Constructor tool vcf2diploid^58^ (version 0.2.4). The haploid genomes were arbitrarily referred to as parent1 and parent2. Trimmed fastq reads were aligned to individual haploid parent genomes using Tophat^40^ and Bowtie2^59^ (version 2.0.6), following a transcriptome-guided approach, setting parameters to acquire the best alignment and allowing up to two mismatches. The alignments acquired for both haploid genomes, for each individual, were merged using the Suspenders tool (version 0.2.3), selecting the single best-quality alignment. IGVTools^60,61^, version 2.3.18, was used to count the reads with one or the other hetSNP allele. Counts of both alleles, C1 and C2, with alleles ordered alphabetically, were used to calculate the adjusted ratio (*C*1 + 0.5)/(*C*2 + 0.5) to measure the ASE effect size. The use of +0.5 as an adjustment was motivated by the predicted reduction in the Taylor-Maclaurin bias estimator. Statistical tests for ASE were carried out via exact tests for departure from binomial expectation under the null hypothesis of (*C*1 = *C*2), using the binom.test() function in R. Tests were only conducted at heterozygous SNP sites where the total number of counts (*C*1 + *C*2) exceeded five reads (defining valid sites). For each sample, we converted the *p* values into FDR using the Benjamini–Hochberg procedure. An FDR threshold of 5% was used to assess ASE significance in the valid sites. ASE signal validation was performed using ASE data generated from previously published lymphoblastoid cell line data^10^. After filtering for SNPs analysed in both data sets (*n* = 54,214) we declared as validated those ASEs discovered in our brain data set that also had an FDR-corrected *p* value of <5% within the lymphoblastoid data set.

### Characterisation of ASE signals

Variant Effect Predictor (VEP)^62^, SNPeff^31^, and SPIDEX^33^ were used to annotate all hetSNPs and to obtain a list of genes that had at least one significant hetSNP. For a subset of ASEs, namely those annotated as protein-truncating variants within VEP (defined as stop gain, donor splice site and donor acceptor mutations) or common across individuals (defined as a ASEs identified in ≥10 individuals), we also investigated the impact of genotype on gene expression. The average expression of the exon or gene (following PEER correction) was calculated separately for individuals homozygous or heterozygous for the variant of interest or if possible across all three genotypes. We then performed *t* tests or ANOVA as appropriate to identify departures from the null hypothesis of no association between expression and genotype.

### Investigation of the disease relevance of ASEs using randomisation

We investigated the enrichment of GWAS risk loci for Parkinson’s disease (with the exclusion of data generated by 23andMe)^1^ and schizophrenia^7^ among ASEs versus non-ASE sites by using a randomisation approach similar to that described by Nicolae and colleagues^34^ to generate an empirical *p* value for the significance of overlaps between risk SNPs and ASE sites, accounting for differences in read depth.

Let *C* be the set of SNPs that are in common between our set of hetSNPs (used for our ASE analyses) and the set of variants available from the GWAS summary statistics of the disease association study. Let *Ase* be the subset of SNPs in *C* declared to be ASE hits in our study, and let *NonAse* be the subset of SNPs in *C* not declared to be ASE hits. For the randomisation procedure, we repeated the following two steps 10^5^ times:

i. Randomly select SNPs with replacement from *NonAse* to create a SNP set with the same size and read depth distribution as that of *Ase*. This was done by placing the *NonAse* into bins based on their average read depth across samples and randomly selecting SNPs from the same to match the average read distribution of the *Ase* set.
ii. Calculate the enrichment of GWAS signals within this SNP set by recording the GWAS *p* values for each SNP within the set and computing the mean of the −log10 transformed values. Let *r*_i_ be the mean value for the ith iteration.

The *z* score for *Ase* (z_Ase_) was obtained using the mean and standard deviation of the *r*_i_ values, and was tested using the pnorm() function in R to obtain a *p* value for the enrichment of GWAS risk SNPs among ASEs versus non-ASEs.

Using a similar approach, we also tested ASEs for evidence of enrichment of eQTLs and SNPs with effects on exon inclusion as predicted by SPIDEX.

### Assessment of brain cell type specificity for eQTLs and ASEs

We used the WGCNA R package^63^ to construct separate gene co-expression networks for substantia nigra and putamen in order to generate modules enriched for cell type-specific gene expression profiles. As input for each network construction, we used genes detected in >70% of samples and with an RPKM of >0.1. Following normalisation using CQN^46^ and accounting for GC content, we used 13 PEER axes (see above) in addition to gender, age, and genetic axes to perform covariate correction. The number of PEER axes was optimised to maximise the clustering of high quality cell markers^64^. We generated signed networks using a soft-thresholding power of 7 for substantia nigra and 8 for putamen networks, which approximated a scale-free topology. We obtained first-pass definitions of the modules via the dynamic Tree Cutting algorithm in WGCNA, and we then refined these modules by applying a k-means algorithm as described in our previous publication^65^. Modules were annotated in terms of cell-specific enrichments using the userListEnrichment R function implemented in the WGCNA R Package, combined with additional enrichment analysis on other marker gene sets^64,66–68^. Consistent with other studies, we robustly identified modules enriched for cell type-specific gene expression profiles^21,66–68^. Genes assigned to modules significantly enriched for brain-related cell type markers and with a module membership of >0.3 were allocated a cell type label of neuron, microglia, astrocyte, oligodendrocyte, and endothelial.

Next, for each eQTL targeting a known genic region, if the target gene was allocated to a cell type then the related eQTL received the same cell type label. In the case of eQTLs targeting unannotated transcribed intergenic regions with high or moderate evidence for association to a known gene, the eQTL received a cell type label based on that known gene’s network position. For eQTLs targeting transcribed intergenic regions with low evidence for association with a known gene or which could not be classified, we assigned the target expression feature to a module (and by inference a cell type) based on its highest module membership (defined as the correlation of expression with the first principal component (“eigengene”) of each module), provided the module membership was at least 0.3. Finally, for each eQTL class and each cell type we applied a Fisher’s Exact test to test for enrichment of that cell type label among the genes associated to the eQTL class, relative to the genes not associated with that eQTL class but with module membership >0.3 to a relevant cell type module. We followed a similar approach for ASEs and assessed ASE-containing genes for significant cell type enrichment.

### Partitioned heritability analysis of ASE sites

GWAS summary statistics were obtained for schizophrenia^7^ and Parkinson’s disease (with the exclusion of data generated by 23andMe)^1^. Stratified LD score regression^67^, a method for partitioning SNP heritability across functional genomic annotations, was used to test for enrichment of schizophrenia and Parkinson’s disease heritability within ASE annotations. Region-specific annotations were defined for putamen and substantia nigra (ASEs with FDR < 0.05 within a brain region were assigned to that region). In addition, an annotation (all) containing all ASEs irrespective of brain region with FDR < 0.05 was defined. Annotations were added individually to the “baseline” model of 53 annotations provided by Finucane et al., which comprises 24 different genome-wide annotations reflecting genetic architecture, such as conserved regions and histone marks. HapMap Project Phase 3 SNPs and 1000 Genome Project European population SNPs were used for the regression and LD reference panel, respectively. Only SNPs with MAF > 5% were used for heritability partitioning and the HLA region was excluded. We report the regression coefficient *p* value, which tests whether the ASE annotations contribute significantly to SNP heritability after controlling for the effects of the “baseline” model.

### Reporting summary

Further information on research design is available in the Nature Research Reporting Summary linked to this article.

## Supplementary information

Supplementary Information File

Reporting Summary

Description of Additional Supplementary Files

Supplementary Data 1

Supplementary Data 2

Supplementary Data 3

Supplementary Data 4

Supplementary Data 5

Supplementary Data 6

Supplementary Data 7

Supplementary Data 8

Supplementary Data 9

Supplementary Data 10

## Data Availability

We have made data available in a web server and via full and summary-statistic data downloads. Using our web resource, http://braineacv2.inf.um.es/, users can access and visualise all forms of transcriptome quantification, eQTLs as well as gene co-expression networks (Supplementary Fig. 1). The RNA-seq, whole-exome sequencing and genotyping data can be accessed through the European Genome-phenome Archive numbers EGAS00001002113 and EGAS00001003065. The source data underlying Figs. 1b, 2b–d, 3a–d, 4b, 5a, b, 6b, c, 7b, and 8a and Supplementary Figs. 2a–c and 3–6 are provided as a Source Data file.

## Source data

Source Data
