# Supplementary material for: Regulatory sites for splicing in human basal ganglia are enriched for disease-relevant information
Source: Nat Commun. 2020 Feb 25;11:1041. doi: 10.1038/s41467-020-14483-x (PMC7042265; doi:10.1038/s41467-020-14483-x)
Supplement: Supplementary file 1 — Supplementary Information File [file 41467_2020_14483_MOESM1_ESM.pdf]

Supplementary Tables

	eQTL PUTM % overlap	eQTL SNIG % overlap
**Brain_Amygdala.v7**	26.5	50.9
**Brain_Anterior_cingulate_cortex_BA24.v7**	37.8	57.3
**Brain_Caudate_basal_ganglia.v7**	48.2	59.9
**Brain_Cerebellar_Hemisphere.v7**	46.2	59.3
**Brain_Cerebellum.v7**	53.3	62.0
**Brain_Cortex.v7**	45.7	61.8
**Brain_Frontal_Cortex_BA9.v7**	40.3	58.6
**Brain_Hippocampus.v7**	30.6	54.7
**Brain_Hypothalamus.v7**	29.7	59.9
**Brain_Nucleus_accumbens_basal_ganglia.v7**	41.9	59.5
**Brain_Putamen_basal_ganglia.v7**	39.3	57.8
**Brain_Spinal_cord_cervical_c-1.v7**	24.7	51.3
**Brain_Substantia_nigra.v7**	19	50.6
**PsychENCODE**	72.5	57.1
**CommonMind**	54.2	55.9

Supplementary Table 1

Table of eQTL replication rates across all GTEx brain-specific eQTL datasets

Region	Relationship to known gene	Number of samples tested in total	Number of samples region validated in	Forward Primer (5’-3’)	Reverse Primer (5’-3’)	Predicted Amplicon (bp)	Representative Sanger Sequence
**DER10633**	High	4	2	CAGATGATGGCCGGTGAAG	CAGGAAGTGTTTGGGATGAGC	229	GTCTGCAATACATATTGCTGTGGGCTGCTGAGCAGACGATAGACAGTGGGGCAGGCTGGCAGCCGTCAGAACAAAAGGGCTTTCTTCTTAAGGTCATTCCTCTTCCAGAGGGCCAGGCGGTCAAACTCCTCGATGCTCATCCC
**DER27107**	High	3	3	GGTTCTGCCTGACATAGACAC	TCAGGTGACAGGAGGAAGC	286	TGTGGAAGGGGAAATTGCATTCATTTAAAGTGTTGATGTATCACCTCCCCAAAACTGTTGGAGTCCTGCCAGATTCTTAAGTGAAGACTGGAGAAAAATCCCCTCATGCATCTGGGTGGGAGTAGCCATTTGGAAATATGCCAGAGCATTCTGTTCTCTTAAAAAGGCCTACTGTTGTGAGATATTGTTTTATCAAACCCTGTTGACCTGGAGGAAGGGAAATATCCAACCCAGCCCTGCAGAGGCTTCCTCCTGTCACCTGAA
**DER28209**	High	7	3	CGCCAATCTCATACAAGCCA	AGGCTATCTTCAGACACGCA	537	CACTGCTGGGATGAATTCACATACACCTGAAAACAGCCCTGACTGGCGGTGGGACTCAGTGCAGGTGGGAAAATGACAAGAAAGTATTGCACAGGAGGAGGAAAGCTGGTGGGGAAGCTATCTCACTTGCAAAGTAAATGGCTTGTATG
**DER1571**	Moderate	6	3	CATTCATTGTCTTCGGAGCCA	TTGCCCAACTGACAACATCTC	321	GTATGGCCTCCACCTTGCTTCTACCTTCCAAGCTCCATTTGTATCTGATTGAAGGAAACCAAACTGCACCTGGCTGCAAGGGGTAAACTTATCTTAGCCAGAGGCATAAAAGACGATCCTTTGCTTCTTAGGGGGAATCTGGGGCTCTTCACTTGATCATCTGAGTCTCTTCATCTGCATCATAAAATTCTTCCTCTTCCTCACTCTCACTCCCTAAAGAGCTCTCCTTGTCCACACACTGTTGGGGGTCCAAAGCCTGTAAAGATAATGCTTCTTCTCCCAGCCTGCGATACGAATACACCTGAAGGTCG

							GAGAATGTTTCTAGCAGCGGACTGTTAAGGGTC
**DER14229**	Moderate	7	4	GAGTGAGGCCGGTTACATTC	GCTTAACCAGTTCCAGGACAC	600	GTTCACCATGTAGAGGACATAGCGCTCAGTCTTATCTTCTGGGACGGAGTCCTCATAGTGAGGGCAGAAAACATCCAGGTAGTCATTGATACAGACATCAATATGGTAGTCACCCCTCTGGAATCTGGGGTTGCTGCTGTTCCAGTAGACAGCGTAGCGGTCGGCGACGGCCTTGGAGCCCGGGTCCTGGCTGAACACACACATCCAGAGCACCAGAAACACCAGCGTCAACATCTCCACGTGCAACATCACGCCTGGCCAGCGGCGGAGCCCCCGACGCGCCACGGGCGGTGCGCCGAGAAGGGTGGGGATCCGGAGCCGCAACCCAGCCTTG
**DER18381**	Moderate	7	1	TCTTCTGCCATTCACTCAGGT	CTATGCGGTTCTTTCAGCCA	412	GCGAGGGGCGGAGGTGATGTGCTAGGCCTGAATTCAAGAGGGGGCGCAGCCACAGGCCCCGCGATGCCGGGGTGGCCTCTTGTAACGACGTTTTAACCGCTGACCTAAGTGGATCCGCTCCTCTCCCGCCGCCCGCTCCCTGCCTGCAGCACAGGATGGCGGCTGGCTTTCCCGAGTGGGCGCAGGATAGCAGCATCAAGGAGCATTGTGTACATGCAGAAGTGCACAGTACCTGGAGTGAAACTGCTTGTGTTCGATTTCTGATACCATTCATAACTGGCTGTGTGATCTCAAAACCTCTAAAATGCAGACCTCCAGCTCTAGATC
**DER15834/5**	Low	4	4	ACGTGTTCCTTGCTGTGTATC	CAGTGGCAGAGTTTGAGAGG	533	TCCTCTCTGAAGAACTCCTGACCAGAAGCTCCCTTTCTGTGTGGGCTAGATTCTTTAACTATTTGACACTTCTTTCATTCTTCTGCAGTTGGTCTGAATGCATTCCTGGGTGAAACAGCTCAGAAGACTGGCTCACTGCAGCAGTGCGTATGCTTCAGGCTGATGCTAAGGCATAACACATCATTGTTGAGATCAGCTCCAAACTGGAACATATTTACTGAGTGAATGGCTTAATGTCTGCTGACTCAT

							GGCTGATTAGGGTGATAACTGCTGAAGGCTCAGGTGGCTGTGGCAATTTCTTAAAACATGACAACACTGAAGTTTGCTGCATCAGTTGATTCTTCCTTTCATGAACTATTTCTCTGTGGTATGTGATGCTGTTTAATAGCATTTTACCCACAGAACTCTCAAAGTTGG
**DER34755/68**	Low	4	4	GAGTTGGAGTTTGCTGTTTCG	AGTGGCTCTTACTCACAGTTTC	274	GTGATTCTCCTGCCTCAGCCACCACCATTTAACACTGAGAAGTGGGCAGTCACAGAGAAAAAGTTTAGAATAGTTCACCAGTGAAGTCATCTCAACCAGCACAGGGCCTTGACTCCTAACAACAATCACACGAACTTGGAAGAGGAGCCTTCCCCAACTGAACCTTCGCTTGAGACCTCGTCCTTGGCCATCCTCTACATCTGGATTCATGATACAGAGAA

Supplementary Table 2

Table of i-eQTL target region validation

Supplementary Figures

a)

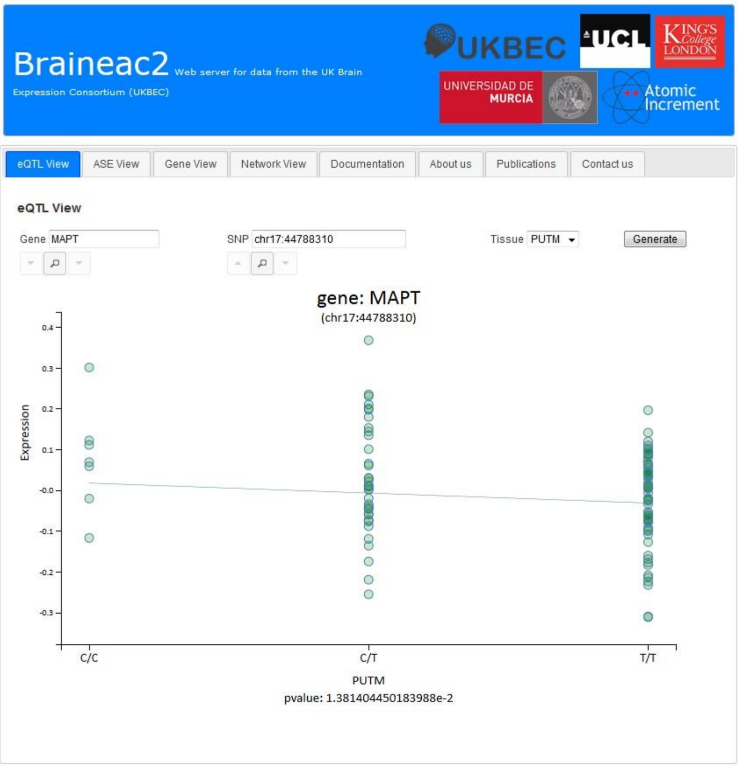

b)

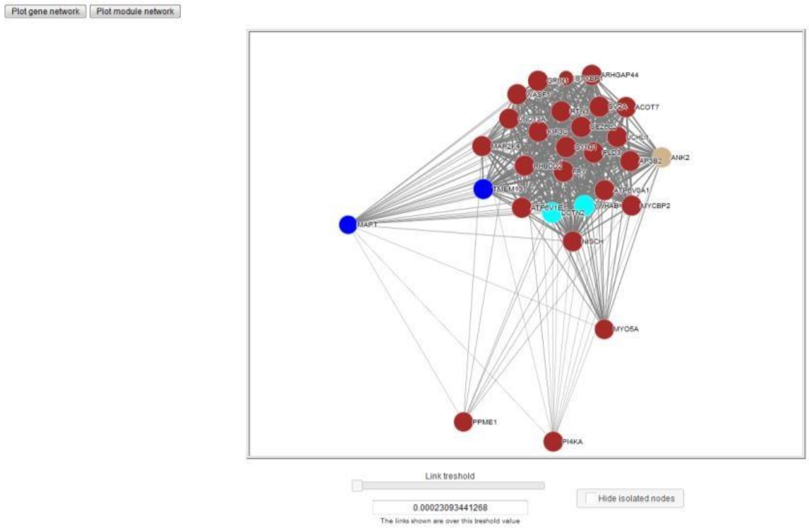

Supplementary Figure 1

Screenshots to show the information accessible through the BRAINEAC web resource

a) Use of BRAINEAC to access eQTL data. b) Use of BRAINEAC to access gene co-expression network data.

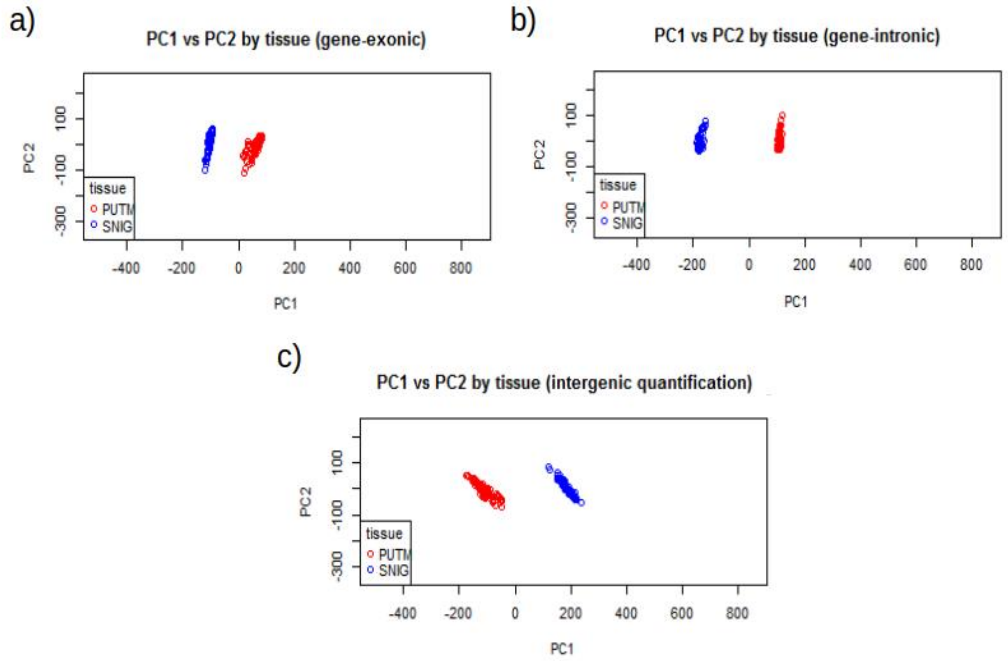

Supplementary Figure 2

Scatter plots to show the first (X-axis) and second (Y-axis) principal components for gene-exonic (a) gene-intronic (b) and transcribed intergenic (c). Samples originated from putamen are coloured in red and samples originated from substantia nigra are coloured in blue. Source data are provided as a Source Data file.

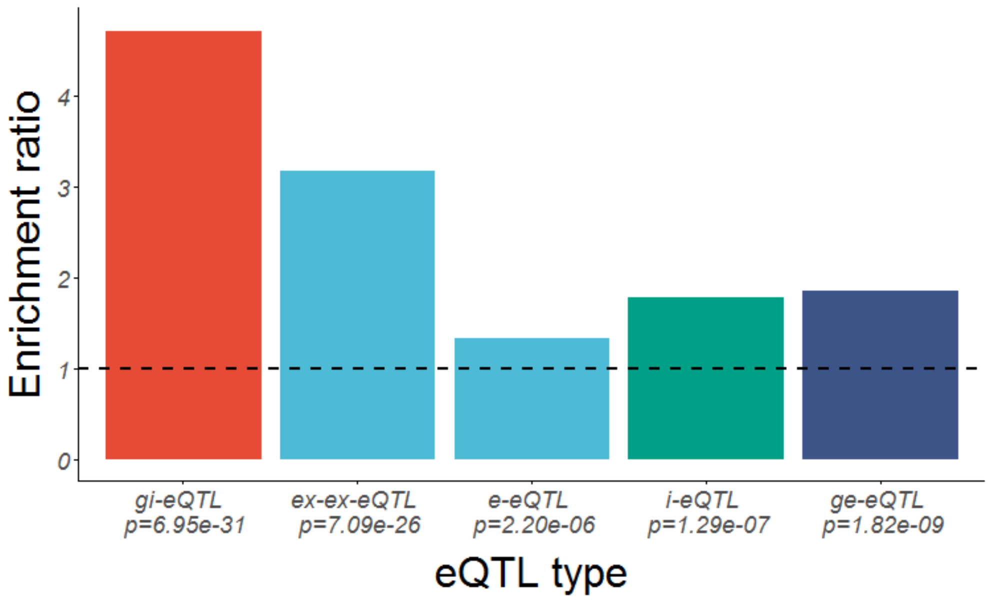

Supplementary Figure 3

Enrichment of risk loci for neurological and behavioural disorders across all eQTL classes. Source data are provided as a Source Data file.

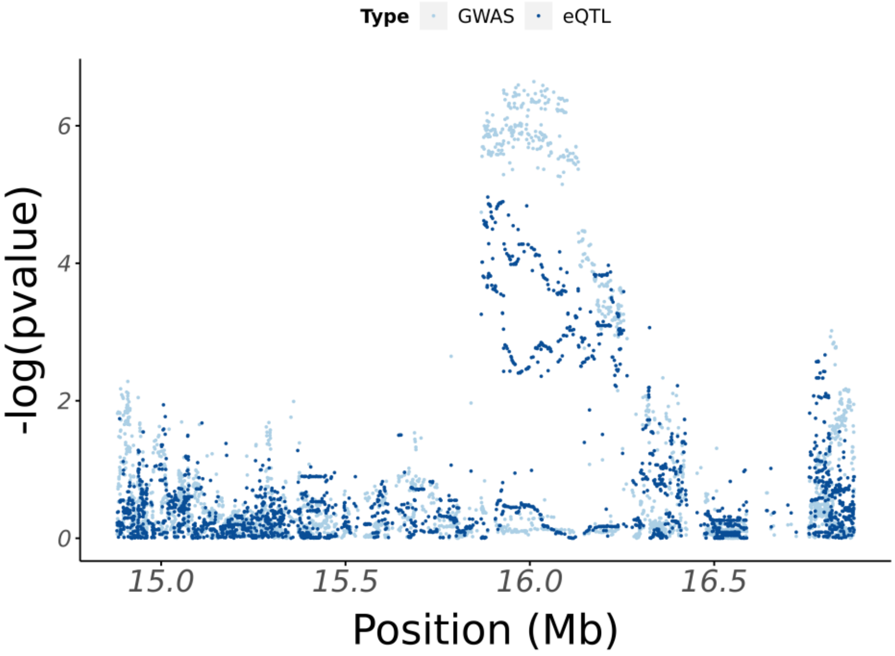

Supplementary Figure 4

Colocalisation of the PD GWAS lead SNP SNP rs4566208 (GWAS p-value = 2.28 x 10^{-7}) and the i-eQTL targeting DER38036 (i-eQTL p-value =1.09 x 10^{-5} in putamen). Source data are provided as a Source Data file.

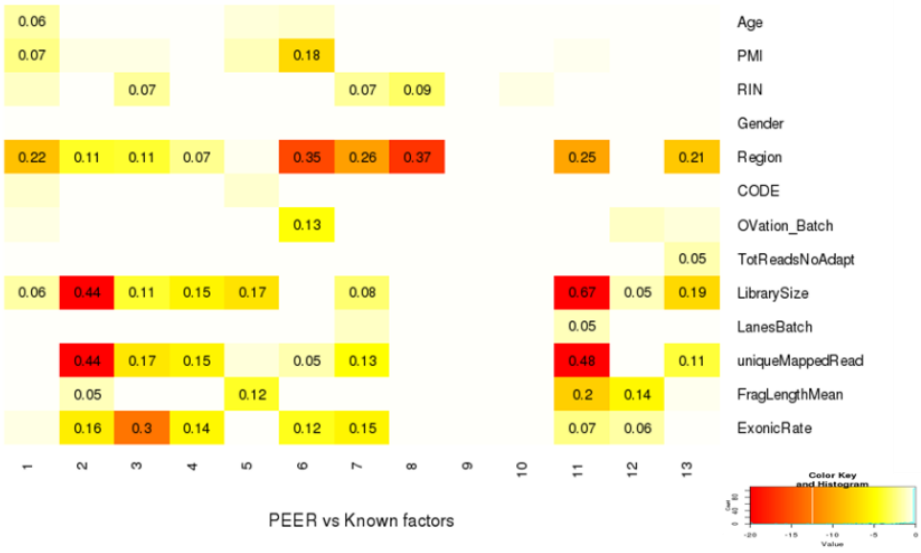

Supplementary Figure 5

Heatmap to show the relationship between PEER axes (X-axis) and known covariates (Y-axis). The FDR-corrected p-values for correlations between each PEER axis and known

factor are depicted by the colour of ea. The Pearson R^2 values are displayed within each cell. Source data are provided as a Source Data file.

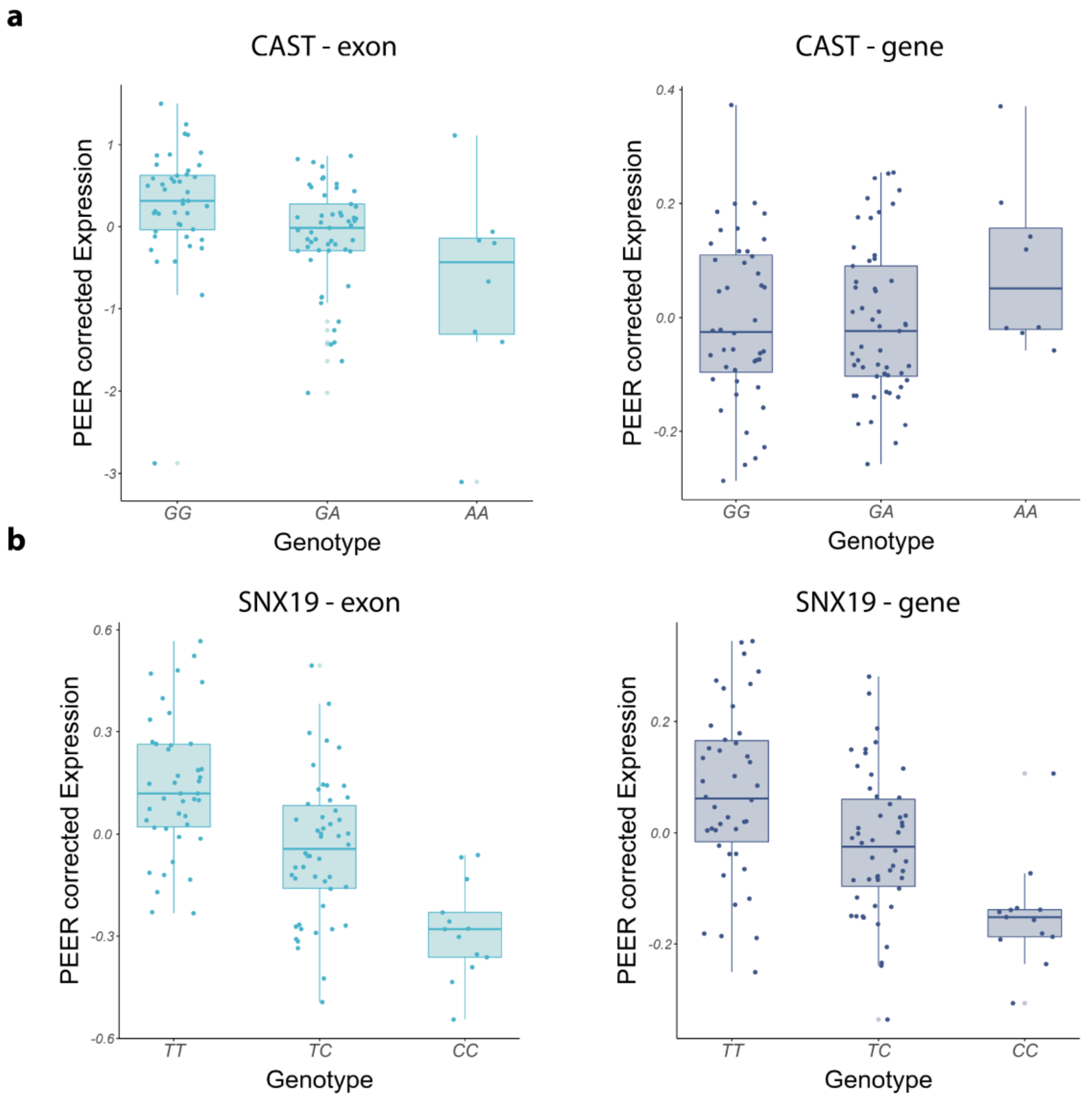

Supplementary Figure 6

Allele-specific expression sites capture both splicing and gene level cis-regulation. (a) Some ASEs appear to operate in an exon-specific manner, and likely represent splicing QTLs. Expression of the exon containing rs7724759, a splice variant present in the *CAST* gene, across individuals of all three genotypes demonstrates that the dosage of the splice variant impacts on exon expression. However, *CAST* gene-level expression across individuals of all three genotypes is unaffected by the dosage of the splice variant. (b) Some ASEs appeared to operate in a gene-level manner. Expression of the exon containing rs1050078, a variant present in the *SNX19* gene, and *SNX19* gene-level expression across individuals of all three genotypes demonstrates a similar dosage relationship. Data presented using Tukey-style box plots. Source data are provided as a Source Data file.
