# Supplementary material for: Regulatory sites for splicing in human basal ganglia are enriched for disease-relevant information
Source: Nat Commun. 2020 Feb 25;11:1041. doi: 10.1038/s41467-020-14483-x (PMC7042265; doi:10.1038/s41467-020-14483-x)
Supplement: Supplementary file 2 — Reporting Summary [file 41467_2020_14483_MOESM2_ESM.pdf]

natureresearch

Corresponding author(s): NCOMMS-18-28841A

Last updated by author(s): Nov 8, 2019

Reporting Summary

Nature Research wishes to improve the reproducibility of the work that we publish. This form provides structure for consistency and transparency in reporting. For further information on Nature Research policies, see Authors & Referees and the Editorial Policy Checklist.

Statistics

For all statistical analyses, confirm that the following items are present in the figure legend, table legend, main text, or Methods section.

n/a	Confirmed	
☐	☒	The exact sample size (*n*) for each experimental group/condition, given as a discrete number and unit of measurement
☐	☒	A statement on whether measurements were taken from distinct samples or whether the same sample was measured repeatedly
☐	☒	The statistical test(s) used AND whether they are one- or two-sided<br>*Only common tests should be described solely by name; describe more complex techniques in the Methods section.*
☐	☒	A description of all covariates tested
☐	☒	A description of any assumptions or corrections, such as tests of normality and adjustment for multiple comparisons
☐	☒	A full description of the statistical parameters including central tendency (e.g. means) or other basic estimates (e.g. regression coefficient) AND variation (e.g. standard deviation) or associated estimates of uncertainty (e.g. confidence intervals)
☐	☒	For null hypothesis testing, the test statistic (e.g. *F*, *t*, *r*) with confidence intervals, effect sizes, degrees of freedom and *P* value noted<br>*Give P values as exact values whenever suitable.*
☒	☐	For Bayesian analysis, information on the choice of priors and Markov chain Monte Carlo settings
☒	☐	For hierarchical and complex designs, identification of the appropriate level for tests and full reporting of outcomes
☐	☒	Estimates of effect sizes (e.g. Cohen's *d*, Pearson's *r*), indicating how they were calculated

Our web collection on statistics for biologists contains articles on many of the points above.

Software and code

Policy information about availability of computer code

Data collection	Human brain samples originating from 117 individuals of European descent were obtained from the Medical Research Council (MRC) Sudden Death Brain and Tissue Bank and the Sun Health Research Institute. All samples were authorised for ethically approved scientific investigation (Research Ethics Committee number 10/H0716/3) and had fully informed consent for retrieval.
Data analysis	For the pre-processing of the data various open-source software were used: Trim Galore!(v0.3.1) to identify and remove adapters, FASTQC(v0.10.1) to assess data quality, tophat2 (v2.0.9) for alignment, HTSeq-Counts5 (v0.5.4p4) for exonic regions, BEDtools (v2.24.0) for intronic regions, DEXSeq (v1.10.6) for individual exons, Altrans (v1.1.02) for exon-exon junctions. Analyses of the processed data were performed with the R software (v.3.1.0).

For manuscripts utilizing custom algorithms or software that are central to the research but not yet described in published literature, software must be made available to editors/reviewers. We strongly encourage code deposition in a community repository (e.g. GitHub). See the Nature Research guidelines for submitting code & software for further information.

Data

Policy information about availability of data

All manuscripts must include a data availability statement. This statement should provide the following information, where applicable:

- Accession codes, unique identifiers, or web links for publicly available datasets
- A list of figures that have associated raw data
- A description of any restrictions on data availability

We have made data available in two primary formats. Using our web resource, http://braineacv2.inf.um.es/, users can access and visualise all forms of transcriptome quantification, eQTLs as well as gene co-expression networks. The RNA-seq, whole exome sequencing and genotyping data can be accessed through the European Genome-phenome Archive numbers EGAS00001002113 and EGAS00001003065.

Field-specific reporting

Please select the one below that is the best fit for your research. If you are not sure, read the appropriate sections before making your selection.

☒ Life sciences ☐ Behavioural & social sciences ☐ Ecological, evolutionary & environmental sciences

For a reference copy of the document with all sections, see nature.com/documents/nr-reporting-summary-flat.pdf

Life sciences study design

All studies must disclose on these points even when the disclosure is negative.

Sample size	This study involved two complementary approaches, both aimed at determining the effect of given genetic variants on gene expression or splicing, namely eQTL mapping and allele-specific expression (ASE). The final number of brain samples used was 170, consisting of 105 putamen and 65 substantia nigra samples.<br><br>The success of previous genotypic gene expression studies, despite the use of modest sample numbers, provided us with empirical evidence for the feasibility of this approach with regional samples originating from approximately 100 individuals. Our own analyses conducted using a similar experimental design showed that a sample size of only n=101 is sufficient to detect cis-acting SNPs capable of influencing gene and exon level expression in human brain (Ramasamy et al., 2014). If we take an extremely conservative approach, and we i) assume that there are on average 12 alternative splicing transcripts per gene and 4 measured exons per transcript, ii) assume all tests are independent, then we calculate study wide significance thresholds of 4e-10 and 5e-11 for transcript level and exon level tests respectively. Combining these thresholds with the estimates of standard error obtained from published data we find that a common expression quantitative trait locus (eQTL) with a 20% minor allele frequency will be detected with 80% power when the per allele effect on the expression level is 20% at the transcript level, or 35% at the exon level. While these represent signals of moderate to high effect, they are in line with the types of eQTL signals already found in medically relevant examples. We are therefore confident that a sample size of n=105 for putamen is adequately powered to detect the moderate to high effects of genetic variation on gene expression.<br><br>This study also included allele-specific expression analysis, which can detect significant effects even with very small sample numbers. This is because with ASE the diploid nature of the human genome is used to measure the ratio of mRNA expression from each allele in the same cellular environment within the same subject. Significant deviations from the expected 50:50 ratio provide the evidence that one allele contains variants that favour higher gene expression. Thus, a major advantage of the method is that the alternative alleles serve as within-sample controls of each other, eliminating environmental influences and measurement biases that can alter the assayed gene expression.
Data exclusions	Removal of individuals likely to be of non-European descent resulted in the exclusion of 3 samples from the analysis. Additionally, we removed 7 samples with less than 15M exonic reads and an exonic mapping rate of less than 10%. We reported the number of individuals excluded. The final number of samples after exclusion is 170, consisting of 105 putamen and 65 substantia nigra samples.
Replication	We used publicly available independent datasets to validate our findings. These independent datasets have been described in full in the Online Methods document within the following sections: "eQTL discovery and replication", "Validation of unannotated transcribed intergenic regions in silico and by Sanger sequencing" and "ASE signal discovery and replication". Furthermore, to validate unannotated transcribed regions we performed Sanger sequencing (described in the Online Methods document in the section entitled "Validation of unannotated transcribed intergenic regions in silico and by Sanger sequencing".
Randomization	Not applicable
Blinding	Blinding was not relevant to this study since this study used post-mortem brain samples alone and measured the impact of genetic variation on gene expression. No treatments of any kind were performed.

Reporting for specific materials, systems and methods

We require information from authors about some types of materials, experimental systems and methods used in many studies. Here, indicate whether each material, system or method listed is relevant to your study. If you are not sure if a list item applies to your research, read the appropriate section before selecting a response.

Materials & experimental systems

n/a	Involved in the study	
☒	☐	Antibodies
☒	☐	Eukaryotic cell lines
☒	☐	Palaeontology
☒	☐	Animals and other organisms
☐	☒	Human research participants
☒	☐	Clinical data

Methods

n/a	Involved in the study	
☒	☐	ChIP-seq
☒	☐	Flow cytometry
☒	☐	MRI-based neuroimaging

Human research participants

Policy information about studies involving human research participants

Population characteristics	All samples originated from individuals reported as of European descent and with no history of neurological disease (checked through detailed neuropathology). Samples were macro-dissected from 88 males and 29 females and the age range at death was 16-85, mean 53.5 (+/- 16.8).
Recruitment	*Describe how participants were recruited. Outline any potential self-selection bias or other biases that may be present and how these are likely to impact results.*
Ethics oversight	Research Ethics Committee number 10/H0716/3

Note that full information on the approval of the study protocol must also be provided in the manuscript.
