# Supplementary material for: Regulatory sites for splicing in human basal ganglia are enriched for disease-relevant information
Source: Nat Commun. 2020 Feb 25;11:1041. doi: 10.1038/s41467-020-14483-x (PMC7042265; doi:10.1038/s41467-020-14483-x)
Supplement: Supplementary file 3 — Description of Additional Supplementary Files [file 41467_2020_14483_MOESM3_ESM.pdf]

Title: Supplementary Data 1

Description: Table summarising brain samples used for the generation of RNAseq data

Title: Supplementary Data 2

Description: Table of gene-intronic eQTLs (gi-eQTLs) at FDR <5%

Title: Supplementary Data 3

Description: Table of exonic eQTLs (e-eQTLs) at FDR <5%

Title: Supplementary Data 4

Description: Table of exon-exon junction eQTLs (ex-ex-eQTLs) at FDR <5%

Title: Supplementary Data 5

Description: Table of gene-exonic eQTLs (ge-eQTLs) at FDR <5%

Title: Supplementary Data 6

Description: Table of intergenic eQTLs (i-eQTLs) at FDR <5%

Title: Supplementary Data 7

Description: Cell type-specific enrichments of eQTL target regions

Title: Supplementary Data 8

Description: i-eQTLs colocalising with GWAS loci for schizophrenia and Parkinson's Disease

Title: Supplementary Data 9

Description: Table of ASEs identified in putamen and substantia nigra with a MAF > 5%

Title: Supplementary Data 10

Description: Enrichment of PD and schizophrenia heritability amongst ASEs using stratified LD score regression
